# Kidney-specific deletion of the BicC family RNA-binding protein 1 triggers an ADPKD-like cystogenic program

**DOI:** 10.1016/j.isci.2026.117020

**Published:** 2026-08-03

**Authors:** Céline Gagnieux, Florian Bernet, Lona Kroese, Romain Guiet, Allison Burns, Roy Combe, Ivo Huijbers, Daniel B. Constam, Benjamin Rothé

**Affiliations:** 1 Ecole Polytechnique Fédérale de Lausanne (EPFL) SV ISREC, Station 19, 1015 Lausanne, Switzerland; 2 MCCA Transgenic Facility, The Netherlands Cancer Institute, Plesmanlaan 121, 1066 Amsterdam, the Netherlands; 3 BioImaging & Optics Platform (BIOP), Faculty of Life Sciences (SV), Ecole Polytechnique Fédérale de Lausanne (EPFL), 1015 Lausanne, Switzerland; 4 BioInformatics Competence Center, Ecole Polytechnique Fédérale de Lausanne (EPFL), 1015 Lausanne, Switzerland; 5 Center of PhenoGenomics, Ecole Polytechnique Fédérale de Lausanne (EPFL), 1015 Lausanne, Switzerland

**Keywords:** ADPKD, polycystic kidney disease, cyst, Bicc1, ARTCyQ, AI, DDX5, p68, YAP, CREB

## Abstract

Autosomal dominant polycystic kidney disease (ADPKD) arises from mutations in polycystin-1/*PKD1* or polycystin-2/*PKD2* that induce injury-repair pathways and dysregulate cAMP and Yes-associated protein (YAP) signaling to promote cyst growth. Cystic kidneys with elevated cAMP levels also develop in mouse embryos lacking the BicC family RNA-binding protein 1 (Bicc1). To prevent embryonic lethality, we flanked *Bicc1* exon 4 with *loxP* sites for conditional knockout (cKO) in renal tubules using inducible Pax8-rtTA-driven Cre in adults, or distal segment-specific deletion *in utero* using Ksp-Cre. Whereas *Bicc1*-deficient adult kidneys formed relatively few cysts within six months, distal nephron-specific deletion induced aggressive PKD-like disease. Automated renal tubule and cyst quantification (ARTCyQ), a novel machine-learning pipeline to analyze immunofluorescent stainings, revealed increasing ambiguity in nephron segment identity and activation of ADPKD-related pathways, including YAP signaling. Accordingly, transcriptional profiling revealed overlap with PKD and injury-repair signatures, supporting a role for Bicc1 in polycystin signaling.

## Highlights

• Timing of Bicc1 loss determines polycystic kidney disease (PKD) severity
• ARTCyQ automates renal tubule and cyst detection and quantification
• The transcriptome of neonatal Bicc1 cKO kidneys overlaps with a PKD signature
• Bicc1 loss increases nuclear YAP, phospho-CREB, and Ddx5 in cyst-lining cells

## Graphical abstract

**Figure:**
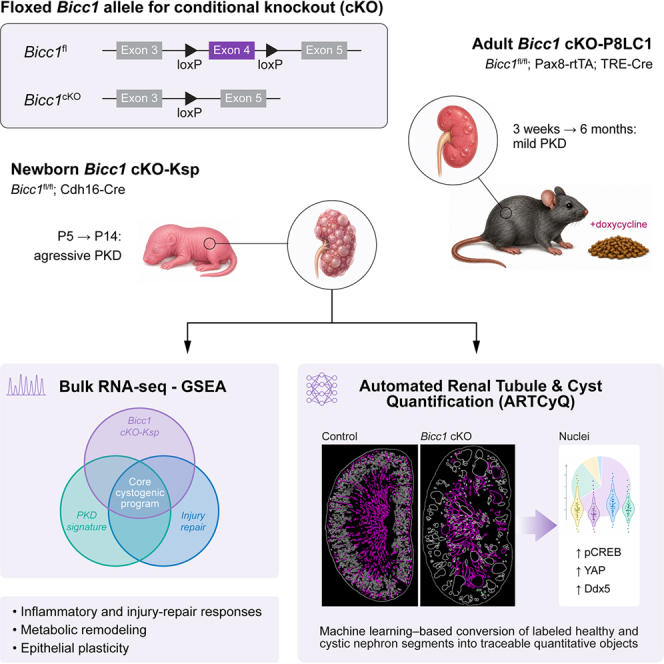

## Teaser

Disease; Pathophysiology; Molecular physiology; Machine learning

## Introduction

Autosomal dominant polycystic kidney disease (ADPKD) is characterized by slowly progressing chronic growth of fluid-filled cysts in both kidneys, with a diagnosed prevalence of 3.3–4.3 per 10,000 individuals in Western populations and accounting for 5% of end-stage renal disease.^1^^,^^2^^,^^3^ In ADPKD, cyst growth involves increased epithelial cell proliferation and transepithelial fluid secretion due to perturbed ciliary calcium signaling, leading to increased cAMP production by the calcium-sensitive adenylate cyclases (Adcy) 5 and 6.^4^^,^^5^ Impaired fatty acid oxidation and altered lipid and glucose metabolism further increase cystic growth.^6^ Under physiological conditions, flow-induced activation of polycystins and Ca^2+^ flux control tubular development and homeostasis by curbing a cilia-dependent cyst activation (CDCA) pathway that appears to converge on the regulation of a cyst-associated mRNA translatome.^7^^,^^8^^,^^9^ However, few known factors can link the regulation of mRNA translation to cilia.

*Bicc1* encodes an RNA-binding protein that was found to be mutated in BALB/c mice with polycystic kidneys (bpk) and in juvenile congenital polycystic kidney (*jcpk*) mice.^10^ Consistently, complete loss of Bicc1 in mouse models results in a PKD-like phenotype,^11^^,^^12^ and human *BICC1* mutations have been identified in patients with cystic renal dysplasia^13^ and ADPKD.^14^ Mechanistically, Bicc1 restrains cAMP signaling by inhibiting the translation of *Adcy6* mRNA and potentially of additional, as yet unidentified transcripts.^11^^,^^15^ Bicc1-mRNA interactions are regulated in *cis* by m^6^A methylation^16^ and in *trans* by Anks3 and Anks6, two ciliopathy-associated proteins.^17^^,^^18^^,^^19^ Anks3 inhibits RNA binding by Bicc1, whereas Anks6 antagonizes Anks3 to restore RNA binding. Accordingly, deletion of *Anks3* in *Pkd1* mutant mice suppresses cyst progression in early- and late-onset ADPKD, consistent with a Bicc1 gain-of-function.^20^ By contrast, Anks6 was found mutated in patients with nephronophthisis and in rodent models with polycystic kidneys.^21^^,^^22^ Besides its role in the kidney, Bicc1 is required downstream of flow-induced polycystin-2 signaling to regulate the expression of the left-right determinant Dand5 during early embryogenesis.^23^^,^^24^ Accordingly, 50% of *Bicc1*^−/−^ mice die *in utero* due to heterotaxia and associated cardiac malformations.^25^ The remaining 50% die within two weeks after birth due to aggressive PKD.^11^ Early postnatal mortality may be confounded by metabolic defects, including decreased renal gluconeogenesis and decreased blood glucose.^26^

To disentangle *Bicc1* functions in kidneys from those in the early embryo and test a role in adults, we flanked exon 4 with *loxP* sites for tissue-specific conditional deletion. In particular, we asked if the pace of PKD progression after *Bicc1* deletion is stage-dependent, and whether cyst-associated signaling pathways and transcriptional alterations overlap with those in orthologous rodent models and in human ADPKD. To address this, we deleted *Bicc1* after embryonic day E11.5 specifically in the distal nephron, or throughout adult renal tubules. Whereas *Bicc1* deletion *in utero* provoked aggressive PKD as expected, kidney cyst burden remained relatively mild for at least six months after *Bicc1* deletion in adults. To comprehensively map cyst localization across nephron segments and the status of known cyst-promoting signaling pathways in the more aggressive disease model, we developed ARTCyQ, a machine learning-based platform that automates detection of renal tubules and cysts and quantifies immunofluorescent signals across entire kidney sections. By combining ARTCyQ with transcriptomic profiling and gene set enrichment analysis, we identify shared pathways and transcriptional overlap between orthologous ADPKD models and *Bicc1*-deficient distal nephron segments that highlight core transcription factors associated with cystogenesis.

## Results

### Bicc1 mRNA is coexpressed with candidate upstream regulators and downstream targets in a nephron segment-specific manner

**Figure 1:**
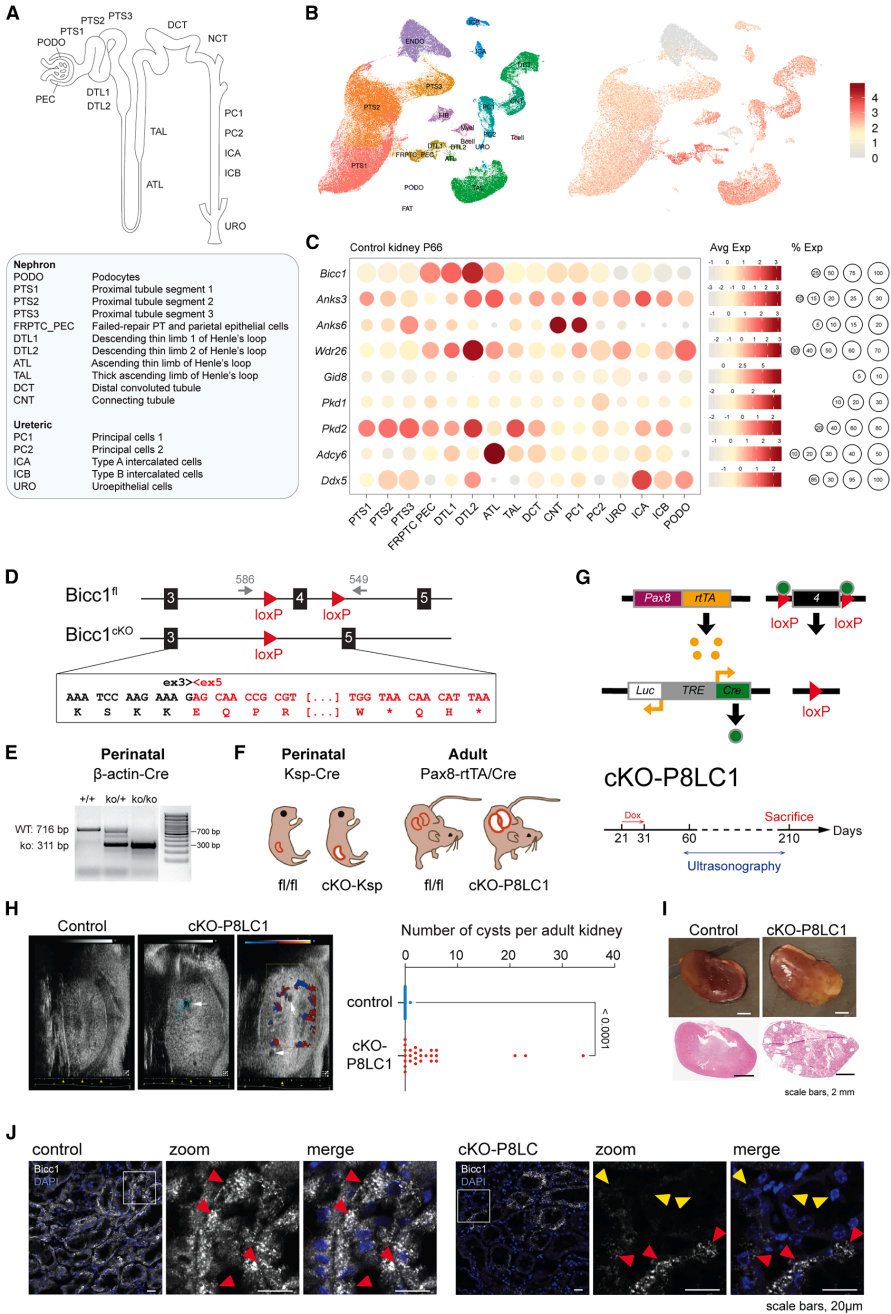
Conditional deletion of a floxed *Bicc1* allele in cKO-P8LC1 mice provokes cyst formation in adult kidneys (A) Schematic representation of the renal epithelial cell types analyzed in B–C and their assignment to nephron or ureteric lineages. (B) t-distributed stochastic neighbor embedding (tSNE) plot of *Bicc1* mRNA expression in adult mouse kidney cell types determined by single-cell RNA-seq analysis. (C) Heatmap of relative mRNA expression levels of the indicated genes of interest across nephron and collecting duct segments of adult mouse kidney. Data in (A–C) are from the Mouse PKD multiome dataset analyzed through the public kidney interactive transcriptomics (KIT) server^28^^,^^29^ (http://humphreyslab.com/SingleCell/). (D) Schematic of the *Bicc1* exon 4 that was flanked by *loxP* sites (flox) using CRISPR/Cas9 editing in mouse zygotes. Excision of exon 4 by Cre recombinase results in a frameshift and premature termination of translation. (E) PCR genotyping of *Bicc1* using primers 586 and 549 in wild-type mice (+/+) and in animals where exon 4 knockout (ko) from floxed alleles was mediated by β-actin-Cre expression in the germline. (F) Schematic depiction of expected kidney growth phenotypes in *Bicc1* cKO mice after crossing with Ksp-Cre or dox-inducible Pax8-rtTA;tetOluciferase/Cre (P8LC1) deleter mice. (G) Experimental timeline for inducible *Bicc1* deletion in cKO-P8LC1 mice. To induce Cre expression, mice received 200 mg/kg doxycycline with the food for 10 days, starting at the age of 21 days. (H) Representative ultrasonography images of kidneys from *Bicc1* cKO-P8LC1 and control littermates, with a cyst highlighted (white arrowhead). The Doppler signal distinguished cysts from arteries (red) and veins (blue). The number of cysts per kidney detected before sacrifice is plotted on the right. *p* value was <0.0001 (Student’s *t* test). (I) Whole-mount images (top) and hematoxylin and eosin (H&E)-stained paraffin sections (bottom) of representative *Bicc1* cKO-P8LC1 and control kidneys of adult mice at the age of 210 days. Scale bars, 2 mm. (J) Kidney sections of adult cKO-P8LC1 and control littermates stained with anti-Bicc1 antibody (white). Magnifications of the boxed areas show the punctate Bicc1 staining alone (zoom) or together with DAPI counterstaining (merge). Red arrows point to tubules expressing Bicc1 while yellow arrows point to Bicc1-depleted tubules. Scale bars, 20 μm.

To investigate if Bicc1 is activated in adult kidneys, we first compared its expression pattern to that of potential regulatory factors and candidate target genes. Healthy postnatal and adult mouse kidneys express Bicc1 protein mainly in proximal tubules (PTs) and in the loop of Henle (LH), and to a lesser extent in collecting duct (CD) cells.^11^^,^^27^ To validate *Bicc1* expression at the mRNA level, we queried scRNA-seq data of adult mouse kidneys in the Mouse PKD multiome dataset through the kidney interactive transcriptomics (KIT) database.^28^^,^^29^ As shown in Figures 1A and 1B, t-distributed stochastic neighbor embedding (tSNE) confirmed the presence of *Bicc1* mRNA mainly in adult nephron and ureteric epithelial cells, with the highest levels accumulating in the LH (Figure 1C). In PTs, *Bicc1* transcription seemed less prominent, contrasting with the relatively intense immunostaining of Bicc1 protein in this segment.^27^ We also analyzed the expression of the Bicc1-regulatory factors *Anks3* and *Anks6*, and of downstream targets such as *Adcy6*, *Ddx5*, and *Pkd2*,^11^^,^^12^^,^^30^ and of *Wdr26*, *Gid8*, and *Pkd1*, which modulate Bicc1 protein levels.^26^^,^^27^ We found that *Anks6* and *Anks3* were co-expressed with *Bicc1* to a variable extent, pointing to potential segment-specific modulation of Bicc1 activity levels (Figure 1C). Furthermore, the C-terminal to LisH complex (CTLH) subunit *Wdr26*, which promotes Bicc1 turnover in cultured IMCD3 cells^26^ was more expressed in distal segments compared to PTs. On the other hand, *Pkd1* expression, which increases Bicc1 protein levels^27^ was detected at a low level in most nephron segments, together with the more variably expressed *bona fide* Bicc1 target mRNAs *Pkd2* and *Ddx5*.^12^^,^^30^ *Adcy6* mRNA was abundantly expressed in the thin ascending limb of LH and in CDs as well, but less in more proximal segments. Variable expression of upstream regulators thus likely modulates Bicc1 activity in a segment-specific manner.

### Bicc1 is required to inhibit cyst formation in adult mouse kidneys

To test if *Bicc1* is required in adult mice, *loxP* sites were inserted 163 or 244 base pairs up- or downstream of exon 4, respectively, using easi-CRISPR editing in C57BL/6J zygotes.^31^ This loxP-flanked “floxed” (fl) exon 4 can be excised by Cre recombinase to generate a conditional knockout (cKO) (Figure 1D). Exon 4 was chosen because of alternative ATG codons in more proximal exons, and because splicing directly to exon 3 will shift the reading frame of exon 5 such that 25 unrelated residues and a premature stop codon will truncate any residual Bicc1 protein after lysine 104 in the first K homology (KH) domain.

After crossing wild-type and *Bicc1*^fl/fl^ or *Bicc1*^fl/+^ mice with an actin-Cre transgene that is active in zygotes,^32^ PCR genotyping confirmed efficient Cre-mediated recombination of the loxP-flanked (fl) allele (Figure 1E). To delete *Bicc1* in adult mice, *Bicc*1^fl/fl^ mice were bred with Pax8; LC1 Cre mice,^33^ where administration of doxycycline induces a Pax8-rtTA transgene that drives Cre recombinase expression specifically in epithelial cells of the genitourinary tract (Figure 1F). Following doxycycline administration with food pellets for 10 days starting at the age of 3 weeks, the resulting *Bicc1* cKO-P8LC1 mice and control littermates were monitored by ultrasonography to non-invasively observe cyst development for 6 months (Figures 1G and 1H), followed by histological analysis at the endpoint (Figures 1I and 1J). 79% of cKO-P8LC1 (*n* = 11/14) but no control animals (*n* = 14) developed kidney cysts. However, only 3 of 27 cKO kidneys examined contained more than 10 macroscopic cysts. Immunofluorescent staining confirmed that Bicc1 was depleted even in most non-cystic tubular epithelial cells, indicating that besides the inhibition of *Bicc1*, a “second hit” is likely required to drive cyst formation (Figure 1J). Since cysts developed slowly and remained infrequent in cKO-P8LC1 kidneys, our subsequent analysis of Bicc1 function in the regulation of cystic growth focused on a new neonatal model.

### Kidney-specific deletion of Bicc1 by Ksp-Cre *in utero* recapitulates PKD-like disease despite sustained Bicc1 expression in PTs

**Figure 2:**
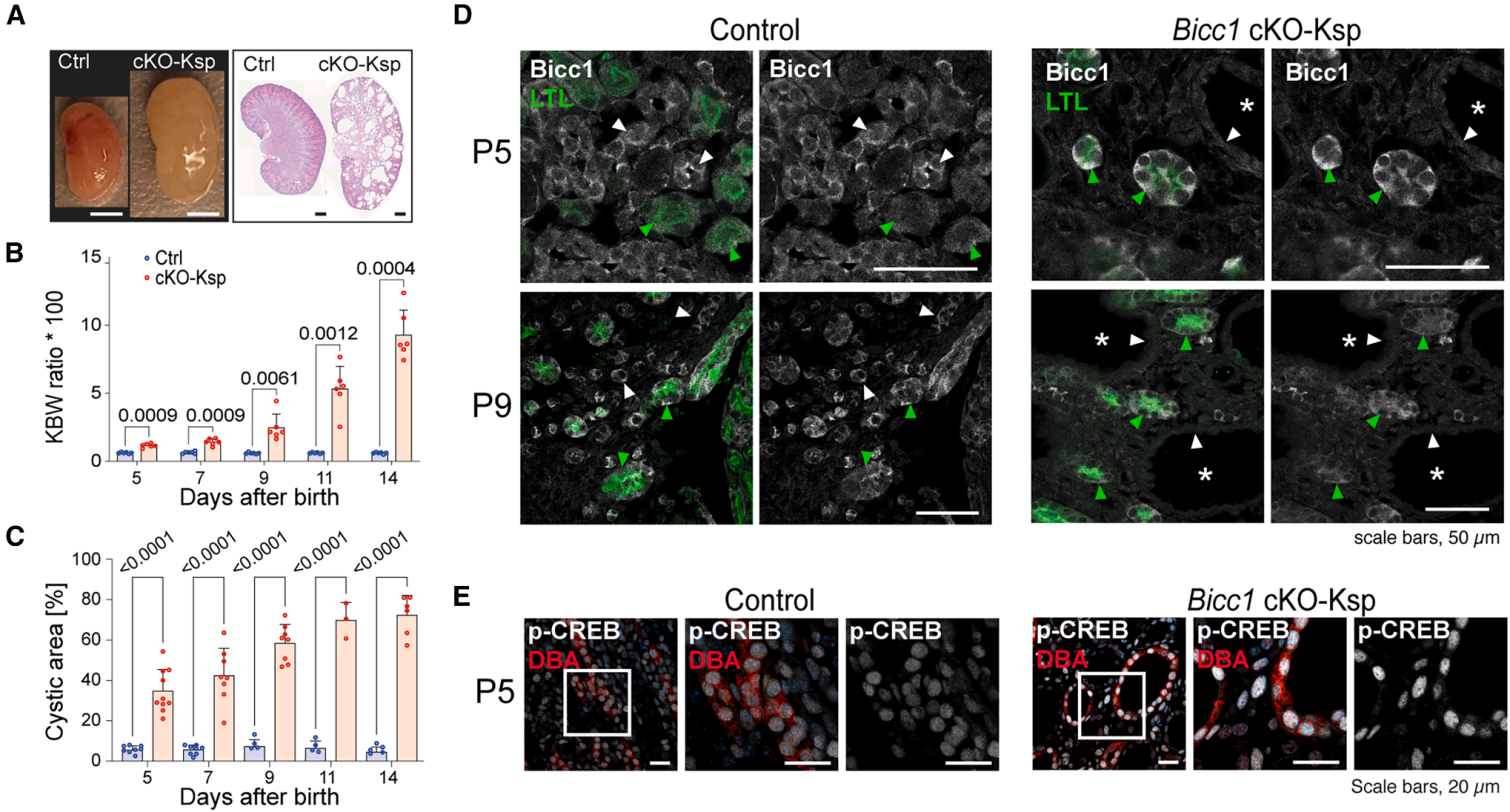
Conditional *Bicc1* knockout *in utero* by Cdh16-Cre (cKO-Ksp) preserves Bicc1 expression in proximal tubules but induces cysts in distal nephron segments (A) Representative *Bicc1* cKO-Ksp and control kidneys and H&E-stained paraffin sections at postnatal day P7. Scale bars, 2 mm and 500 μm, respectively. (B) Kidney to body weight (KBW) ratio of control and *Bicc1* cKO-Ksp pups examined at the age indicated. Data are means +SD from 6 animals per genotype at each stage. *p* values (exact numbers) are shown above (Student’s *t* test). (C) Relative cystic area normalized to the total kidney area of 35 *Bicc1* cKO-Ksp and 29 control littermates. Data are means +SD from 3 to 10 animals per time point. All *p* values were <0.0001 (Student’s *t* test). (D) Cryosections of *Bicc1* cKO-Ksp and control kidney that were stained with anti-Bicc1 antibody (white) and with lotus tetragonolobus lectin (LTL, green) at postnatal days P5 or P9. White arrows indicate LTL^−^ segments that were depleted of Bicc1 and formed cysts (asterisks). Green arrows indicate LTL^+^ proximal tubules that retain Bicc1 expression and did not form cysts. Scale bars, 50 μm. (E) Cryosections of *Bicc1* cKO-Ksp and control kidney that were stained with anti-pCREB antibody (white) and with the collecting duct marker dolichos biflorus agglutinin (DBA, red) on postnatal day P5. The magnified boxed areas show pCREB staining together with DBA (middle panels) or individually (right panels). Nuclei are counterstained with DAPI (blue). Scale bars, 20 μm.

*Bicc1* null mutants frequently die *in utero* due to heterotaxia defects.^25^ Backcrossing to an inbred C57BL/6J background for >10 generations further reduced the survival of live pups from at least 14 to only 2–4 days after birth. To circumvent this precocious mortality, we crossed *Bicc1*^fl/fl^ mice with the kidney-specific (Ksp) transgene, where a *Cdh16* promoter fragment drives Cre expression in distal tubules and CDs, starting at embryonic day E11.5^34^ (Figure 1F). *Bicc1*^fl/fl^; *Cdh16*-Cre (cKO-Ksp) mice developed no left-right asymmetry defects and survived at normal Mendelian frequency until at least postnatal day P14 even on an inbred C57BL/6J background (*n* = 30/131). Nevertheless, kidney sizes were palpably enlarged in the *Bicc1* cKO-Ksp mutants on both sides already at postnatal day P5. Average kidney-body weight (KBW) ratios were 2-fold increased at that stage compared to control littermates, and 10-fold by day P14, correlating with the presence of numerous large fluid-filled cysts in histological sections (Figures 2A and 2B). Accordingly, the average cystic area relative to H&E-stained tissue surface was enlarged from <0.1 in control P5 kidneys to 0.3 in cKO-Ksp and continued to linearly increase to 0.7 by P14 (Figure 2C). Thus, *Bicc1* cKO-Ksp kidneys develop aggressive PKD-like disease without pre- or early postnatal lethality.

Immunofluorescent staining of frozen kidney sections confirmed Bicc1 protein depletion in cKO-Ksp kidneys, except in PTs where Cdh16-Cre is not active and which are stained by fluorescent Lotus tetragonolobus lectin (LTL) (Figure 2D).^34^ In addition, we monitored the phosphorylation of the transcription factor cAMP responsive element binding protein 1 (CREB) on Ser133 that can be induced by cAMP/PKA.^35^ Immunofluorescent staining of phospho-CREB appeared to be increased specifically in nuclei of cyst-lining cells, at least in CDs marked by red fluorescent Dolichos biflorus agglutinin (DBA) staining (Figure 2E). To validate this conclusion, we decided to quantify immunofluorescence stainings systematically across whole sections of multiple kidneys by machine learning.

### Development of automated renal tubule and cyst quantification (ARTCyQ) as a machine-learning tool to analyze cysts and their spatial distribution

To automate the identification of nephron segments with or without cysts and to quantify changes in protein expression in large immunostaining datasets, we developed a machine learning algorithm named automated renal tubule and cyst quantification (ARTCyQ) (Figure S1). The shapes of tubules and cysts are not similar enough to detect them by the same morphological criteria. Therefore, ARTCyQ employs two complementary workflows. Tubules are detected and classified using the deep neural network Omnipose^36^ that we trained on the nephron segment-specific markers LTL for PTs, DBA for CDs, Tamm-Horsfall (THP)/uromodulin for the ascending limb of the loop of Henle (LH) (aLH), and sodium–chloride cotransporter (NCC) sodium-chloride cotransporter for distal convoluted tubules (DCTs) (Figures S1A and S1B). By contrast, cysts can be identified by a random tree pixel classifier based on nuclear 4',6-diamidino-2-phenylindole (DAPI) staining alone, followed by spatial mapping of the cyst-lining epithelial cells. Cysts that stain positive for segment markers are then classified accordingly (Figures S1A and S1C). In a final step, tubules and cysts are each segmented into cytoplasmic and nuclear components. Thereafter, fluorescent signal intensities and morphological features are extracted for each object (i.e., a single tubule or cyst) and its cytoplasmic and nuclear compartments. Each object is also assigned a unique identifier, enabling bidirectional tracing between the original image and the processed dataset.

### ADPKD-related signaling pathways are differentially deregulated in Bicc1 mutant cyst-lining cells in different nephron segments

**Figure 3:**
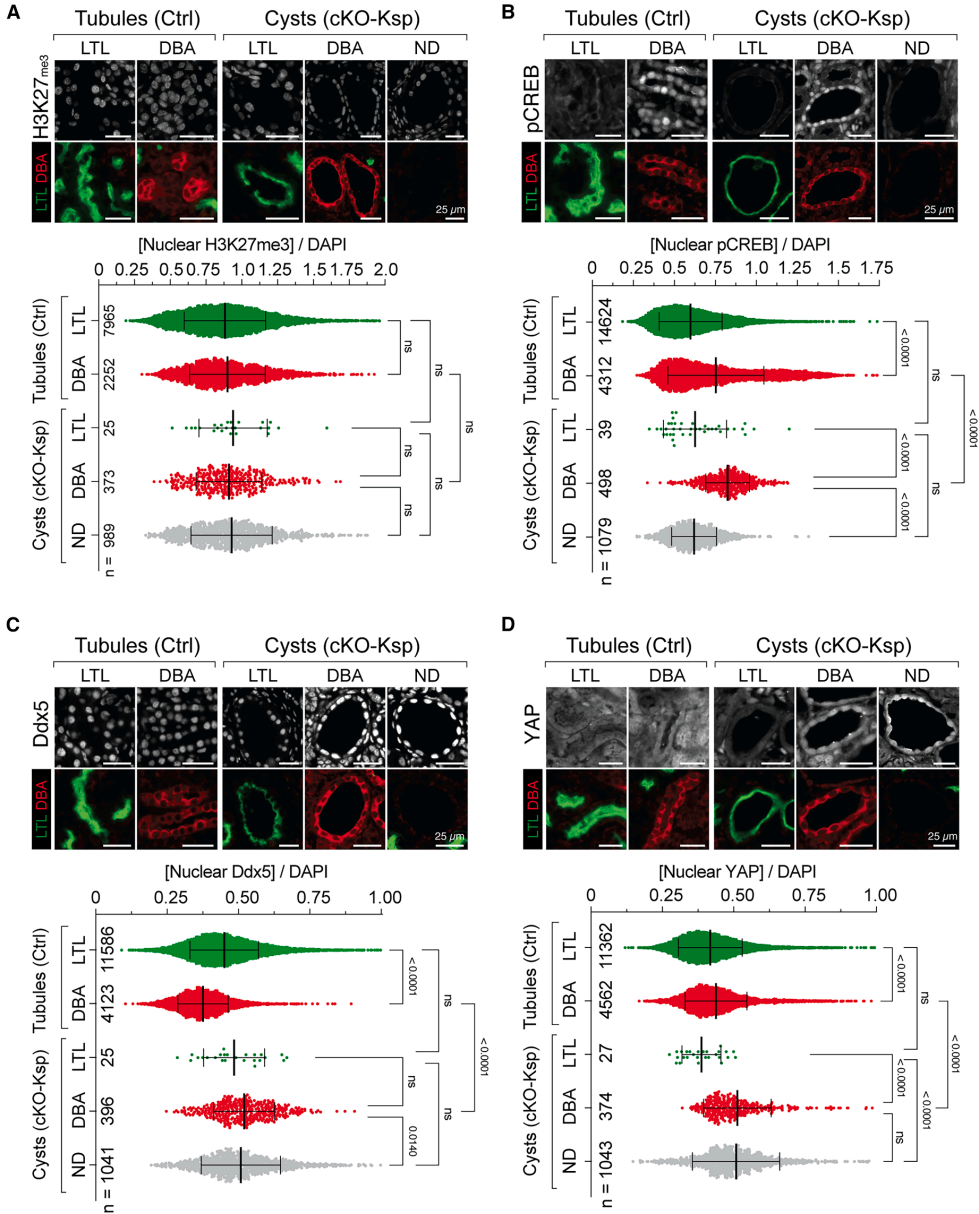
Bicc1 deletion increases nuclear pCREB, Ddx5, and YAP in cyst-lining collecting duct cells Representative immunofluorescent images of (A) H3K27me3, (B) pCREB, (C) Ddx5, and (D) YAP (white) in kidney sections of *Bicc1* cKO-Ksp mice or control littermates at postnatal day P5, counterstained with LTL (green) and DBA (red) to mark PTs and CDs, respectively. ND, not determined. Below, nuclear fluorescence in each of the indicated classes of objects was normalized to DAPI for signal quantification. Each data point represents an individual tubule or cyst, with signal intensities averaged across all nuclei within that object. n, number of objects. H3K27me3 was used as a control. Due to non-normal data distribution and unequal sample sizes, statistical analysis was performed using the Kruskal-Wallis test followed by Dunn’s post-hoc test for multiple comparisons. Data are presented as mean ± standard deviation (SD) from at least 4 control and 6 cKO-KSP animals. Exact *p* values are indicated in the graphs; ns, not significant. Scale bars, 25 μm.

To spatially map and quantify cysts and immunostainings of pCREB and other direct or indirect candidate targets of Bicc1, we tasked ARTCyQ to compare whole kidney sections from *Bicc1* cKO-Ksp animals with those of control littermates at postnatal day P5 (Figure 3; Table S1). Owing to the restricted number of fluorescence channels, we labeled only PTs and CDs. Cysts in unstained segments were classified as “not determined” (ND). Immunofluorescent staining of the heterochromatin marker H3K27me3 was similar across all objects in cKO-Ksp and control kidneys, confirming that cysts did not influence the signal quantification by ARTCyQ (Figures 3A, S2, and S7). By contrast, nuclear pCREB staining in control kidneys was on average 1.26-fold stronger in CDs than in PTs (Figures 3B, S3, and S7). In cKO-Ksp, this difference increased to 1.32-fold due to elevated pCREB levels in CD cysts. In cystic and non-cystic PTs, and in cysts classified as ND, nuclear pCREB staining remained low. These findings indicate that loss of Bicc1 increases CREB activation in CDs but not in PTs, consistent with a possible increase in cAMP/PKA signaling.

*Bicc1*^−/−^ kidneys contain elevated levels of both Adcy6 protein and cAMP.^11^ *Adcy6* mRNA is clearly transcribed in CDs (Figure 1C), but antibodies to analyze Adcy6 protein levels in CDs by immunostaining are lacking. As an alternative candidate target, we stained the RNA helicase Ddx5, whose 3′UTR has been shown to mediate translational repression by Bicc1 in *Xenopus* embryos.^30^ Western blot analysis confirmed that Ddx5 protein levels were increased in *Bicc1* cKO-Ksp kidney extracts (Figure S4A). Consistent with a post-transcriptional mechanism, a luciferase reporter mRNA containing the proximal 3′UTR of murine *Ddx5* was repressed in HEK293T cells by co-transfected Bicc1 (Figures S4B and S4C). Furthermore, endogenous *Ddx5* mRNA co-immunoprecipitated with endogenous Bicc1 in IMCD3 cells specifically if these were depleted of Anks3 by doxycycline-inducible shRNA to facilitate access of specific target mRNAs to the Bicc1 KH domain repeat^18^ (Figures S4D–S4F). Together, these data corroborate that Bicc1 inhibits Ddx5 expression by binding the proximal 3′UTR. In control kidneys, nuclear Ddx5 levels were 20% higher in PTs than in CDs (Figure 3C, S5, and S7). Interestingly, in cKO-Ksp neonates, nuclear Ddx5 levels increased by 39% specifically in CD cyst-lining cells. Ddx5 also accumulated at similarly high levels in cysts classified as ND. These results indicate that Ddx5 expression is broadly derepressed in nephron segments lacking Bicc1.

Transcriptional regulators that promote cystic growth in patients with ADPKD and in orthologous mouse models include Yes-associated protein (YAP).^37^^,^^38^ Immunostaining of *Bicc1* cKO-Ksp kidney sections revealed a significant increase in nuclear YAP in cyst-lining cells of CDs (+17%) (Figures 3D, S6, and S7). ND cysts exhibited similarly elevated nuclear YAP levels, whereas cystic PTs showed no difference relative to control PTs. These results corroborate that kidney-specific deletion of *Bicc1* in cKO-Ksp mice induces ADPKD-like changes in distal nephron segments.

### ARTCyQ analysis reveals dynamic changes in cyst burden and epithelial cell identities along distal nephron segments

**Figure 4:**
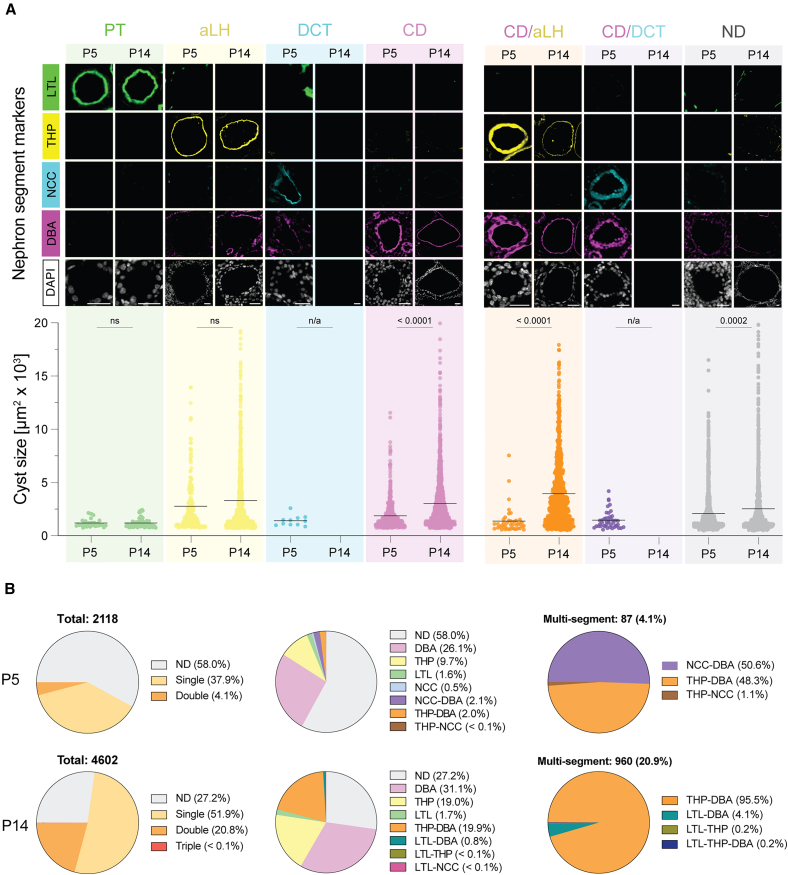
Automated quantification of dynamic changes in cyst sizes and their nephron segment identities over time in *Bicc1* cKO-Ksp kidneys (A) Representative cysts stained with nuclear dye (DAPI, bottom row) and the indicated nephron segment markers in kidney sections of *Bicc1* cKO-Ksp mice at postnatal days P5 or P14. Fluorescent signals in each channel are shown separately. Scale bars, 25 μm. Below, the frequency and size distribution of individual cysts per nephron segment were quantified using ARTCyQ to automatically identify and bin single, double, or triple positive cysts, and cysts of non-determined origin (ND). For cysts that were positive for multiple segment markers, only classes representing ≥2% of all cysts in at least one age group are shown. Data represent the means from 10 cKO-KSP animals at P5, and from 8 at P14. Exact *p* values (Student’s *t* test) are indicated in the graphs; ns, not significant; na, not applicable. (B) Pie charts of the relative proportions of cysts that stained positive for the indicated nephron segment markers at P5 or P14. Left: Proportions of cysts positive for none (ND), one, or two to three segment markers. Center: Detailed distribution of all cysts. Right: Relative frequency of cysts lined by cells that stained positive for at least two segment markers at the same time.

To determine the distribution of cysts along renal tubules and whether it changes with time, we tasked ARTCyQ to quantify and compare dilated nephron segments at postnatal days P5 (10 animals) versus P14 (8 animals). Individual cysts were measured to plot their lumenal area and classified into groups expressing either several of the segment markers examined, or only one, or none (Figure 4; Table S2). Between P5 and P14, the average surface area of individual cyst lumina increased both in CDs and in the ND category (Figure 4A). A similar trend was observed in the aLH, although it did not reach statistical significance (*p* = 0.0735). By contrast, the rare dilations in DCTs and PTs representing only ≤2% of all single-segment cysts disappeared or remained small (<2,000 μm^2^). Since we observed no deletion of *Bicc1* in PTs (Figure 2D), PT cysts may be induced by pressure from neighboring cysts^39^ but likely fail to grow owing to the presence of Bicc1. By contrast, DCT dilations may adopt a perturbed segment identity. In line with this notion, many cysts (58%) expressed none of the segment markers examined (Figure 4B). More importantly, while only 38% of all cysts were exclusively positive for a single marker corresponding mainly to DBA (26%) and, to a lesser extent, THP cysts (10%), a sizable fraction (4%) co-expressed DBA together with THP or with the DCT marker NCC (Figure 4B). Interestingly, all cyst-lining cells in these cases typically coexpressed both segment markers simultaneously rather than in a mosaic pattern (Figure 4A). By P14, the proportion of such dual identity cysts increased 5-fold to reach 21% (mostly THP^+^/DBA^+^). The proportions of cysts with no known segment identity (27%) or belonging uniquely to CD (31%) or aLH (19%) significantly changed as well. Consistent with *Pkd1/Pkd2* mouse models in which cysts can arise along the entire nephron, including CD, *Bicc1* cKO-Ksp cysts predominantly form in CDs and LH, while the scarcity and subsequent disappearance of NCC-positive cysts suggests progressive loss of DCT marker expression.

### Gene expression changes in newborn Bicc1 cKO-Ksp kidneys significantly overlap with those described in human ADPKD

To profile transcriptional changes associated with cystic growth, we subjected total RNA from *Bicc1* cKO-Ksp kidneys on postnatal day P5 to bulk RNA-seq analysis. Male and female kidneys were analyzed separately (*n* = 4 mice of each sex per genotype, 1 kidney per mouse). Across samples, 4–6 x 10^7^ sequencing reads were obtained per kidney, with no overt outliers or over-represented RNA, adapter, or vector sequences, indicating a good quality of the samples and sequencing reads (Figure S8A). Furthermore, 80–90% of the reads showed unique alignments to the mouse genome mm10 (Ensembl 102, November 2020, 55,487 annotated genes; Figures S8B and S8C). Alignments on other genomes were limited mainly to human.

**Figure 5:**
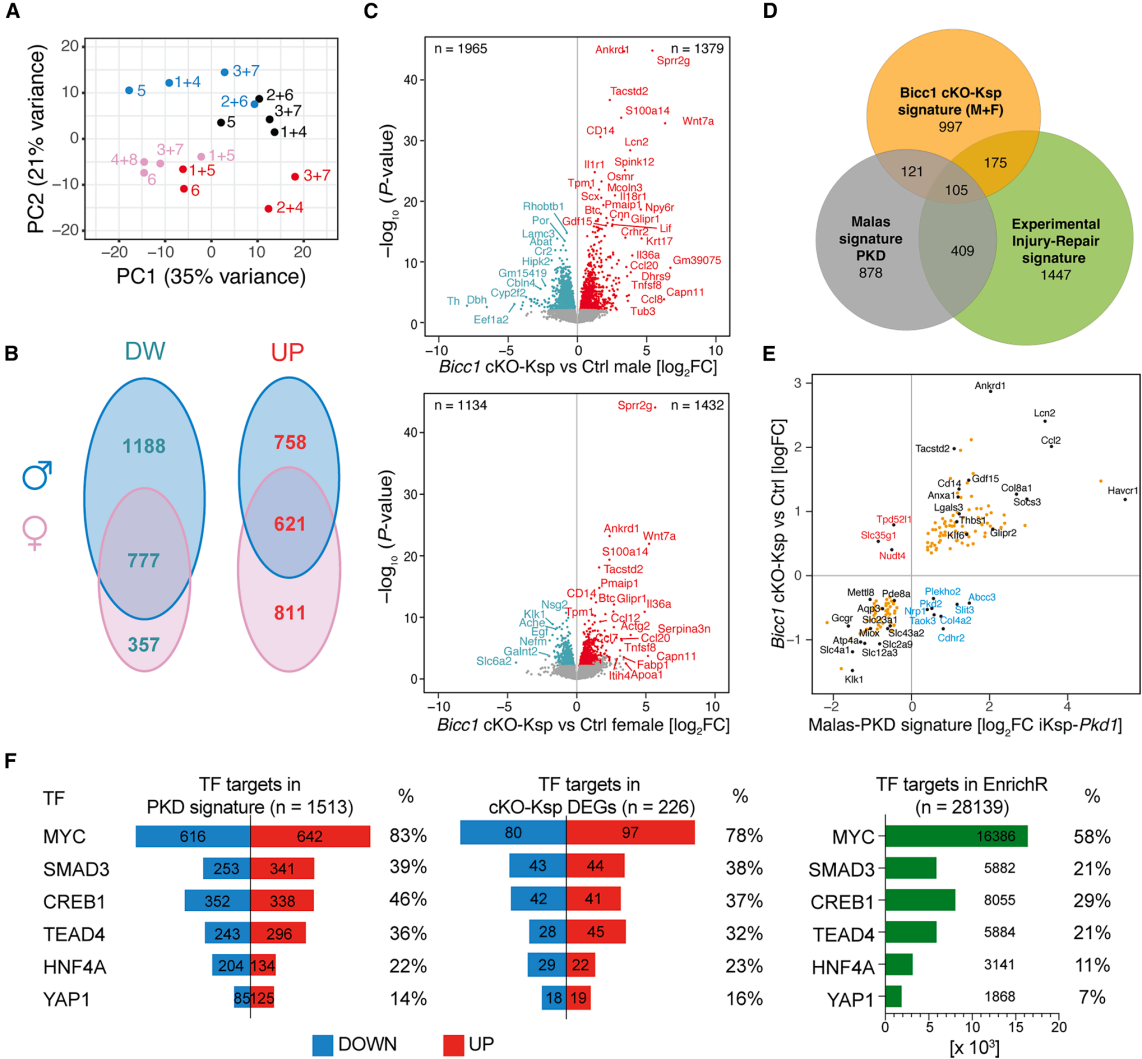
Gene expression changes in newborn *Bicc1* cKO-Ksp kidneys overlap with human PKD and injury-repair transcriptional signatures (A) Principal component analysis (PCA) of bulk RNA-seq data from postnatal day 5 (P5) *Bicc1* cKO-Ksp and wild-type control (Ctrl) kidneys. Samples segregate primarily by genotype along PC1 (35% of total variance) and by sex along PC2 (21% of total variance). Each dot represents one kidney (*n* = 4 mice per sex and genotype). (B) Numbers of significantly downregulated (DW) and upregulated (UP) genes in *Bicc1* cKO-Ksp versus sex-matched Ctrl kidneys (*p* < 0.05), shown separately for males (blue) and females (pink). Overlapping regions indicate genes deregulated in both sexes in the same direction. (C) Volcano plots show differentially expressed genes (DEGs) in male (top) and female (bottom) *Bicc1* cKO-Ksp kidneys compared with sex-matched controls. Log_2_ fold change (log_2_FC) is plotted against −log_10_(*p* value). Significantly upregulated genes are shown in red and downregulated genes in blue; selected genes of interest are labeled. (D) Venn diagram shows overlap between genes deregulated in the same direction in both male and female *Bicc1* cKO-Ksp kidneys, and in the experimental injury-repair (EIR) or PKD signatures described by Malas et al.^40^ Numbers indicate shared and unique genes across datasets. (E) Comparison of log_2_FC values for genes shared between the *Bicc1* cKO-Ksp and Malas-PKD signatures. Genes displaying discordant regulation between datasets are indicated in blue and red. (F) Transcription factor binding site (TFBS) enrichment analysis. Left, distribution of predicted TFBS among up- and downregulated genes in the Malas-PKD signature. Middle, TFBS among the 226 *Bicc1* cKO-Ksp DEGs shared between sexes and with the PKD signature. Right, total number and percentage of genes associated with each TF in the unified EnrichR library. Colors indicate downregulated (blue) and upregulated (red) targets.

Principal component (PC) analysis showed that *Bicc1* cKO-Ksp and wild-type control (Ctrl) littermate samples were separated primarily by the first PC accounting for 35% of the variance, whereas sexes were separated mainly by the second PC accounting for 21% of the variance (Figure 5A). Pairwise comparisons between cKO kidneys and sex-matched controls showed significant upregulation of 1379 genes in males and 1432 genes in females, and downregulation of 1965 genes in males and 1134 genes in females (*p* < 0.05). The number of genes that were up- or down-regulated in both sexes, typically in the same direction, was 622 and 777, respectively (Figures 5B and 5C; Table S3). A complementary analysis including sex as a covariate confirmed that genotype-associated transcriptional changes were largely sex-independent (Figures S8D–S8F; Table S3). Among these 622 up-regulated genes, Kyoto Encyclopedia of Genes and Genomes (KEGG) pathway analysis revealed that the most enriched gene ontology (GO) terms included cytokine-cytokine receptor interaction, cell cycle, and apoptosis, among others, whereas the most enriched terms among the 777 down-regulated genes were metabolic pathways, bile secretion, peroxisome proliferator-activated receptors (PPAR) signaling pathway, PT bicarbonate reclamation, and retinol metabolism (Table S4). These changes are consistent with the notion that likely ADPKD drivers include inflammation and changes in cell cycle, metabolism, and cell survival signaling.

To further probe the relevance of *Bicc1* mutant mice for human PKD, we compared transcriptional changes in *Bicc1* cKO-Ksp kidneys to a PKD signature derived from a previous meta-analysis of de-regulated genes in patients with ADPKD, and in rodent models, including *Pkd1*-deficient mice.^40^ Interestingly, among all 1398 genes that were de-regulated in the same direction in both male and female *Bicc1* cKO-Ksp kidneys (*p* < 0.05), 226 (16%) were shared by this Malas-PKD signature. 105 of these 226 genes (46%) also overlapped with an experimental injury repair (EIR) signature described by the same study^40^ (Figure 5D; Table S5). Together with 175 genes that do not belong to the PKD signature but which are nonetheless deregulated in EIR models, these 105 transcripts amount to 20% of all *Bicc1*-regulated genes, consistent with the observed KEGG pathway alterations and with the view that sustained activation of a repair program in response to tissue injury and inflammation likely instigates cystic disease.^41^

Most of the 226 *Bicc1*-regulated genes that overlap with the PKD signature changed their expression in the same direction in both datasets (Spearman r = 0.82, *p* < 2.2e−16), including the highly upregulated genes *Lcn2, Tacstd2, Ccl2, Cd14, Havcr1*, and *Gdf15,* which are already implicated in kidney injury^42^^,^^43^^,^^44^^,^^45^^,^^46^^,^^47^^,^^48^ (Figure 5E; Table S5). Furthermore, upregulation of *Il18r1* and downregulation of *Taok3* may function upstream by promoting stress-induced inflammation.^49^^,^^50^

### Overlap between Bicc1-regulated genes and the PKD signature highlights core transcription factors associated with cystogenesis

**Figure 6:**
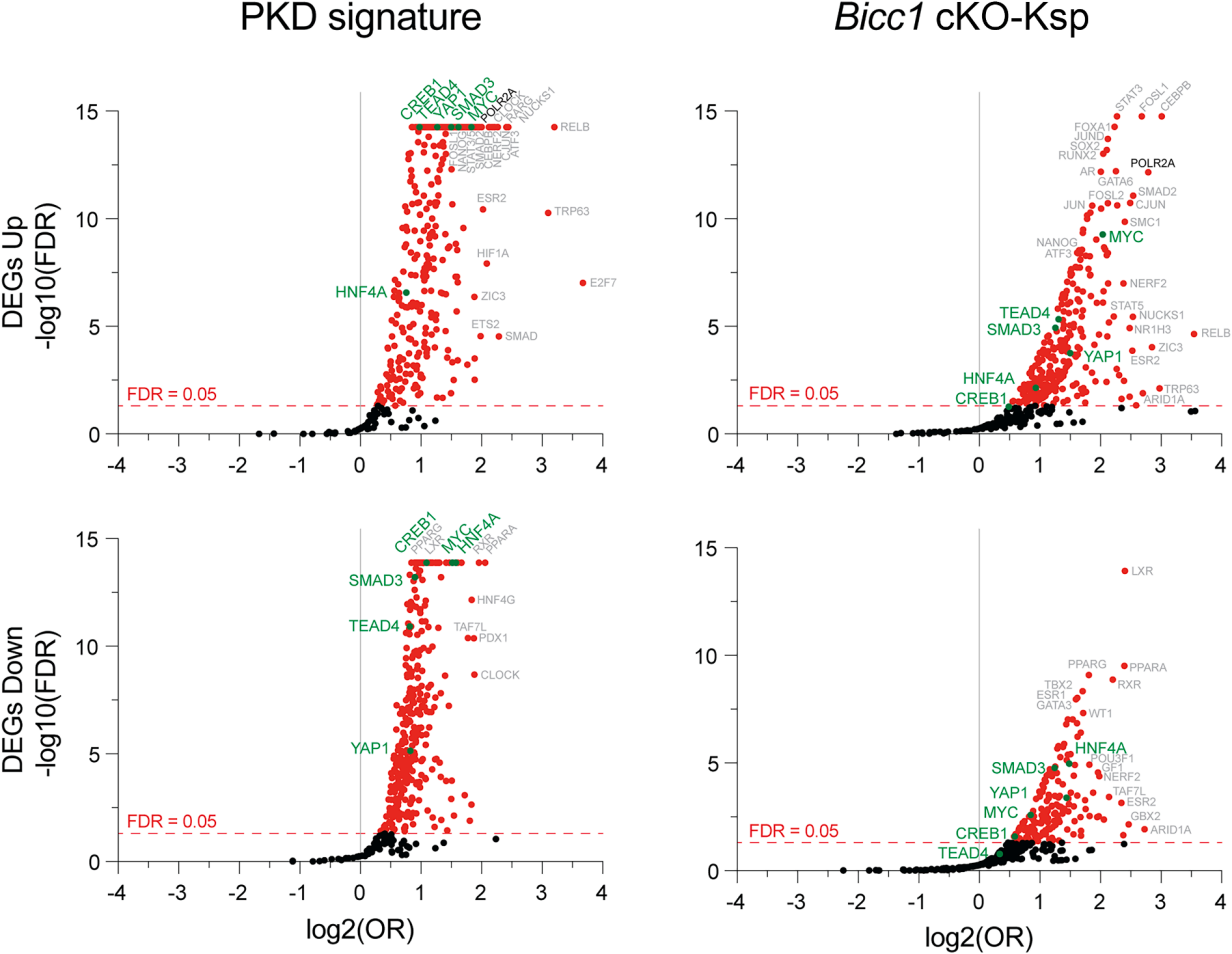
Enrichment of transcription factor binding sites in the PKD signature and in *Bicc1*-regulated PKD-associated genes Volcano plots show the enrichment or depletion of transcription factor (TF) binding sites among differentially expressed genes (DEGs) in the Malas-PKD signature (left) and in the subset of 226 *Bicc1* cKO-Ksp DEGs that overlap with the PKD signature (right). Analyses are shown separately for upregulated (top) and downregulated DEGs (bottom). Each point represents a TF, plotted according to the log_2_ odds ratio (OR; x axis) and −log_10_ (false discovery rate, FDR; y axis). Odds ratios quantify the relative likelihood that DEGs are targets of a given TF compared with non-DEGs. OR > 1 indicates enrichment, OR < 1 indicates depletion. Statistical significance was assessed using Fisher’s exact test with Benjamini-Hochberg correction for multiple testing. The horizontal dashed line indicates the significance threshold (FDR = 0.05). Significantly enriched or depleted TFs are shown in red or blue, respectively. Selected PKD-relevant TFs are highlighted in green, while additional highly significant TFs are shown in gray. For visualization purposes, FDR values equal to zero were set to the smallest non-zero FDR observed. PKD-relevant TFs highlighted in green showed the following enrichment values in the *Bicc1* cKO-Ksp DEG subset: MYC (OR = 4.1; FDR< 0.0001); CREB1 (OR = 1.4; FDR = 0.0534); SMAD3 (OR = 2.4; FDR <0.0001); TEAD4 (OR = 2.5; FDR <0.0001); YAP1 (OR = 2.8; FDR = 0.0002); HNF4A (OR = 1.9; FDR = 0.0072). Additional TFs discussed in the main text also showed significant enrichment, including CJUN (OR = 5.6; FDR <0.0001), STAT5 (OR = 2.3; FDR = 0.0002), RELB (OR = 11.7; FDR <0.0001), NANOG (OR = 3.1; FDR <0.0001), TRP63 (OR = 7.9; FDR = 0.0077), LXR (OR = 3.0; FDR <0.0001), PPARα (OR = 2.4; FDR = 0.0041). OR and FDR values associated with each TF in each experimental condition are available in Table S8.

To identify transcriptional regulators associated with cyst formation, we analyzed transcription factor binding site enrichment in genes shared between the *Bicc1* cKO-Ksp signature and the Malas PKD signature. To focus on a library of experimentally validated TF-target interactions, we integrated all ChIP-seq-based resources of the EnrichR server into one^51^ (Table S6). Interestingly, binding sites of known PKD-relevant TFs, including MYC proto-oncogene (MYC), CREB1, SMAD3, TEAD4, YAP1, and HNF4A,^52^^,^^53^^,^^54^ were similarly enriched above their prevalence in the genome both among differentially expressed genes (DEGs) of *Bicc1* cKO-Ksp kidneys and in Malas-PKD signature genes. For example, among the 226 PKD signature genes that are regulated by *Bicc1*, this enrichment ranged from 16% (37/226) for YAP1 to 78% (177/226) for MYC (Figure 5F; for a more comprehensive list of TFs, see Table S7). Binding sites of several additional TFs that are implicated in PKD-related processes were also enriched among upregulated DEGs in both signatures, including CJUN, whose activation by c-Jun N-terminal kinase (JNK) correlates with cyst burden,^55^ STAT5, which promotes abnormal proliferation in ADPKD,^56^ and RELB proto-oncogene (RELB), which is overexpressed in cystic rat and human kidneys^57^ (Figure 6; Table S8). Furthermore, recent gene set enrichment analysis (GSEA) in *Pkd2* mutant mice revealed a similar enrichment of homeobox transcription factor Nanog (NANOG) and TRP63 target genes.^58^ In contrast, TFBSs enriched in downregulated DEGs were primarily nuclear receptors involved in lipid metabolism, including retinoid X receptor (RXR)/liver X receptor (LXR) and PPARα. While RXR/LXR signaling is also altered in ANKS6 R823W mutant cystic rat kidneys,^59^ decreased expression of PPARα in PKD mouse models contributes to cyst progression.^60^^,^^61^ Together, these findings indicate that deleting *Bicc1* stimulates transcriptional programs of inflammation, injury repair, cell proliferation, and metabolic alterations, delineating a core regulatory network associated with renal cystogenesis.

## Discussion

In this study, kidney-specific deletion of a new conditional *Bicc1* allele, combined with AI-driven quantitative imaging and transcriptional profiling, identified new stage-dependent roles for *Bicc1* in renal tubule homeostasis. Whereas *in utero* deletion using Ksp-Cre uncoupled aggressive cystic disease from the embryonic mortality seen in *Bicc1*-null mutants, doxycycline-inducible *Bicc1* deletion after weaning using Pax8LC1 induced only sparse and slowly growing cysts. Furthermore, analysis of Bicc1-depleted kidneys during development revealed that cyst formation involves signaling pathways shared with ADPKD and partial overlap with an established PKD gene signature, including enrichment of shared transcription factor binding sites. Therefore, genes that are similarly deregulated in PKD and in this novel non-orthologous disease model likely represent a core transcriptional program of cystogenesis.

### Bicc1 is required to prevent kidney cyst formation in adult mice

Previous studies established that constitutive *Bicc1* mutations and null alleles provoke an aggressive PKD-like disease already during development.^10^^,^^11^^,^^12^ By crossing a new conditional *Bicc1* allele with Pax8LC1 Cre deleter mice, we here found that inducible *Bicc1* deletion after weaning is sufficient to trigger cystogenesis. However, these adult kidneys rarely developed more than 10 cysts before the age of 6 months, precluding us from conclusively profiling associated molecular changes or cyst origins at that stage. Interestingly, a delay in disease onset has also been reported in other PKD models after weaning, including in mice where *Pkd1* or *Pkd2* were inactivated only after postnatal day 14.^58^^,^^62^^,^^63^ Nevertheless, in these orthologous disease models, the cyst burden at the age of 6 months clearly exceeds that which we observed in Bicc1-depleted adult kidneys, suggesting that some polycystin functions are likely Bicc1-independent. Alternatively, a formal possibility is that Bicc1 function is context-dependent, inhibiting cyst formation when polycystins are functional but promoting it when they are mutated. Of note, cilia also both inhibit and promote cyst formation.^64^ Future studies should test if Bicc1 has a similar dual role.

### Uncoupling Bicc1-dependent cystogenesis from confounding metabolic effects

The sharp decline in cyst formation after terminal renal maturation pointed to a developmental switch coinciding with the transition from feeding on milk to a less fatty, solid diet. In *Pkd1*-deficient mice, this transition has been linked to the cessation of tubular epithelial proliferation.^62^^,^^63^^,^^65^ Consistent with a role for fatty acids in modifying disease progression, *Pkd1*-deficient cells exhibit impaired fatty acid oxidation, and dietary fat restriction ameliorates cystic disease severity.^66^ In *Bicc1* null mutant mice, a substantial fraction of cysts originates from PTs,^11^^,^^12^ a segment responsible for renal gluconeogenesis and where energy supply involves fatty acid oxidation. In mice that lack Bicc1 in all nephron segments, including PTs, metabolic rewiring is thus likely a confounding factor to further aggravate cystogenesis.

### Characterization of the Bicc1 cKO-Ksp model as an orthogonal approach to identify epithelial-intrinsic cyst drivers

To preserve Bicc1 expression in PTs, we specifically targeted distal nephron segments and CDs using Ksp-Cre. *Bicc1* cKO-Ksp mice were born at Mendelian frequency with no laterality defects or overt extra-renal abnormalities and survived throughout the 14-day postnatal window analyzed. Nevertheless, *Bicc1* cKO-Ksp kidneys recapitulated the aggressive cystic disease described in *Bicc1*^bpk/bpk^ and null mutants, concurring with the conclusion of a recent report that used another conditional *Bicc1* allele that was generated independently.^67^ In addition, we found that kidney enlargement and cyst expansion were accompanied by the activation of canonical ADPKD-associated signaling pathways in targeted segments, including increased nuclear pCREB and YAP. Consistently, loss of Bicc1 also leads to the upregulation of urinary markers of kidney injury that predict ADPKD severity, such as α-fetuin, Cystatin C, and neutrophil gelatinase-associated lipocalin (NGAL).^68^^,^^69^ Collectively, these observations establish *Bicc1* cKO-Ksp mice as a model to analyze PKD-relevant mechanisms of cystogenesis in the absence of PT cysts and associated metabolic changes as likely confounding factors of disease severity and/or early mortality.

### The intersection between Bicc1 and PKD transcriptional signatures highlights core transcriptional regulators of cyst formation

Only 16% of transcriptional changes in *Bicc1* cKO-Ksp kidneys overlapped with a previously established PKD signature.^40^ Some differences likely reflect the fact that our dataset captures an earlier postnatal stage (P5) than the established PKD signature (P12-P14, and beyond). On the other hand, the overlap between these distinct signatures helps to pinpoint a core transcriptional program associated with cyst formation across disease models and developmental stages.

Among the most strongly upregulated transcripts were the kidney injury markers *Havcr1* and *Lcn2*. Both are similarly upregulated in ADPKD, with *Lcn2* being implicated in promoting glycolysis by inducing mitochondrial dysfunction via mTOR pathway activation.^42^^,^^43^^,^^70^ Other top upregulated genes included the chemokine CCL2, which recruits CCR2-positive leukocytes, and the pro-inflammatory TLR4 agonist Cd14. Expression of CCL2 correlates with cyst expansion and early mortality in human ADPKD and promotes PKD progression in *Pkd1*-deficient mice.^46^^,^^48^^,^^71^ Cystogenesis in ADPKD also correlates with the upregulation of CD14, consistent with a role for inflammation in promoting PKD progression.^45^

Transcription factor binding site enrichment analysis of the shared gene set revealed strong enrichment for PKD-relevant transcriptional regulators, including MYC, CREB1, YAP/TEAD, SMAD3, and HNF4A. Upregulation of YAP targets such as Ankrd1 and Col8a1 is consistent with increased nuclear YAP in cyst-lining cells and supports a role for mechanotransduction in Bicc1-dependent cyst expansion.^37^ Additional PKD-relevant TFs include STAT5, RELB, TRP63, and NANOG, which regulate cell proliferation, stress responses, and epithelial plasticity.^56^^,^^57^^,^^58^ In contrast, downregulated genes were enriched for nuclear receptors such as RXR/LXR, PPARα, and HNF4α, consistent with metabolic perturbations in ADPKD.^59^^,^^60^^,^^61^ Together, these findings indicate that cyst formation across distinct cystic kidney disease models converges on a restricted transcriptional network orchestrating inflammatory and injury-repair responses, epithelial plasticity, and metabolic remodeling.

### ARTCyQ as a methodological advance for renal tubule and cyst analysis

To allow unbiased cyst and tubule mapping directly from immunofluorescence (IF)-stained kidney sections, we developed the deep learning algorithm ARTCyQ. Unlike existing approaches that segment renal cysts at the organ scale using CT or MRI,^72^^,^^73^ or which perform automated segmentation in kidney histology,^74^ ARTCyQ is designed to operate on immunofluorescence microscopy images, enabling high-resolution, whole-section analysis at the cellular and structural level. A major strength of ARTCyQ lies in its ability to perform quantitative cyst mapping in an automated and reproducible manner. By minimizing user intervention and subjective selection of regions of interest, the method reduces observer bias and enables robust comparisons across experimental conditions. Importantly, detection parameters can be tuned to balance sensitivity and specificity according to experimental needs. The present study used stringent detection settings to minimize false positives. A detailed training pipeline that can be efficiently retrained and transferred across datasets and experimental contexts further increases the adaptability of ARTCyQ beyond the present application (see Materials and Methods for access to the code). Overall, ARTCyQ provides a versatile platform that can be readily extended to human kidney tissue, including biopsy and autopsy samples, thereby opening new avenues for translational and clinical research.

### Ddx5 as a potential Bicc1-regulated factor in polycystic kidney disease

ARTCyQ analysis revealed that loss of Bicc1 increases nuclear levels of PKD-relevant TFs YAP and pCREB in cyst-lining cells. Consistent with their activation, binding sites for YAP and CREB were significantly enriched among dysregulated genes identified by GSEA. In parallel, we examined the status of Ddx5, previously identified as a target of Bicc1-mediated translational silencing in *Xenopus*.^30^ Ddx5, also known as p68, is an adenosine triphosphate (ATP)-dependent RNA helicase belonging to the DEAD-box protein family, which is overexpressed in most human cancers and contributes to tumor progression.^75^ Depending on the cellular context, Ddx5 functions as either a transcriptional coactivator or corepressor. Notably, it potentiates β-catenin activity to promote the expression of target genes such as c-Myc or c-Jun,^76^^,^^77^ and it directly enhances MYC transcription by resolving G-quadruplex structures.^78^ Interestingly, c-Myc has previously been implicated in cyst progression downstream of YAP,^37^ which potentially links Ddx5 upregulation with a critical signaling module of ADPKD.

Elevated Ddx5 levels have also been detected in cyst-lining epithelial cells from human patients with ADPKD.^79^ Importantly, *Ddx5* mRNA levels are not increased in *Bicc1* cKO kidneys and are absent from the PKD transcriptional signature. This uncoupling between transcript and protein abundance strongly suggests translational regulation by Bicc1, consistent with its established molecular function^80^ and prior observations in *Xenopus*.^30^ The strong sequence conservation between the proximal 3′UTR of *Ddx5* in mouse and *Xenopus laevis* (∼72% identity across the 271 proximal nucleotides), together with our luciferase reporter assays and RNA co-immunoprecipitation analyses, further supports *Ddx5* mRNA as a conserved Bicc1 target.

### ARTCyQ points to an erosion of cell identity associated with disease progression

ARTCyQ uncovered progressive alterations in the expression of cyst segment markers during disease progression. In the *Bicc1* cKO-Ksp model, early cysts predominantly form in CDs and the aLH, consistent with the Cre expression pattern. Over time, however, an increasing proportion of cysts display mixed segment marker staining, most notably THP^+^/DBA^+^ dual positivity. The homogeneous distribution of these markers throughout entire cysts argues against simple fusion events and instead may reflect altered epithelial states or lineage plasticity changes. Loss of NCC expression further illustrates this phenomenon. In line with possible alterations in cell identities, single-cell transcriptomic analysis of human ADPKD kidneys and multiomic single-nucleus profiling of *Pkd1* mouse kidneys revealed unexpected heterogeneity among cyst-lining epithelial cells, uncovering multiple transcriptional states that transcend classical nephron segment boundaries.^29^^,^^81^ Interestingly, a recent lineage-tracing study reports that Cre-driven activation of YAP in distal nephron segments disrupts epithelial identity, with loss of DCT and connecting tubule (CNT) markers and non-cell-autonomous downregulation of PT genes.^82^ This apparent erosion of cell identities accompanies enhanced cell proliferation, disruption of epithelial architecture, and induction of injury-associated genes, including Havcr1 and Lcn2. YAP-associated epithelial plasticity might therefore contribute to deregulating the expression of segment markers in Bicc1 mutant cysts. Future studies using lineage tracing and single-cell approaches will be necessary to directly assess the plasticity of cyst-lining epithelial cells and determine whether Bicc1 keeps YAP and other pathways in check to maintain renal tubule cell fates.

In mouse kidneys lacking polycystin-1 or polycystin-2, cystic growth depends on the presence of functional primary cilia.^64^ By unleashing the CDCA pathway, *Pkd1* mutant kidneys dramatically increase the translation of *Anks3* mRNA as a critical driver of cystic growth.^8^^,^^20^ Interestingly, Anks3 acts as a negative regulator of Bicc1.^17^^,^^18^ Thus, Bicc1 and its regulation by Anks3 emerge as strong candidates to mediate post-transcriptional control of gene expression by the CDCA pathway. Future studies combining ARTCyQ with spatial proteomic approaches will enable direct mapping of protein-level changes and *in situ* assessment of cystogenic signaling pathways at cellular resolution.

### Limitations of the study

Compared to the aggressive PKD-like disease observed in *Bicc1* cKO-Ksp neonates, cyst formation after Bicc1 loss in adult cKO-P8LC1 mice remained rather limited and progressed only slowly for at least six months. Only the cKO-Ksp kidneys were thus suitable for developing the ARTCyQ pipeline, which requires sufficient cystic burden to enable meaningful quantitative analysis. Similar characterization of cysts in adults would require large cohorts of aged mice, which we could not ethically justify.

The main limitation of ARTCyQ is that cytoplasmic compartments are defined by DAPI-negative regions as a proxy, rather than by a direct readout. Segmented objects delineated as tubules thus include the tubule lumen, leading to a slight underestimation of the mean cytoplasmic fluorescence intensities. Similarly, in objects segmented as cysts, the quantification of cytoplasmic fluorescence intensities could be slightly biased by the fact that ARTCyQ defines the cyst-lining epithelial compartment as the surface between the apical side facing the cyst lumen and the basal side that is assigned by a fixed distance. Importantly, however, this limitation does not affect tubule or cyst classification because stringent marker-intensity thresholds prevent their misassignment. Furthermore, this study only quantified absolute fluorescence intensities of nuclear pCREB, Ddx5, and YAP, rather than of cytoplasmic proteins. Since ARTCyQ identifies nuclei directly by DAPI staining both in cysts and healthy tubules, nuclear fluorescence measurements are unlikely to be biased by boundary inaccuracies in either of these objects. Future versions of ARTCyQ could improve cytoplasmic segmentation by incorporating a dedicated cytoplasmic marker into the classifier, thereby enabling more accurate delineation of cytoplasmic regions.

## Resource availability

### Lead contact

Further information and requests for resources and reagents should be directed to and will be fulfilled by the lead contact, Benjamin Rothé (benjamin.rothe@epfl.ch).

### Materials availability

All reagents generated in this study are available from the lead contact with a completed Materials Transfer Agreement.

### Data and code availability

ARTCyQ workflow, scripts, and model training are publicly available at Zenodo: https://doi.org/10.5281/zenodo.19066950. RNA-seq data have been deposited in the Gene Expression Omnibus (GEO) database (GEO: GSE337067). In-house analysis code can be found at Zenodo: https://doi.org/10.5281/zenodo.21070463.

## Acknowledgments

The authors would like to thank Dr. Bastien Mangeat (EPFL Gene Expression Core Facility) and Dr. Christian Iseli from the EPFL-UNIL Bioinformatics Competence Center (BICC) for help with generating and analyzing bulk RNA-seq data, respectively; Dr. Edith Hummler (University of Lausanne) for providing Pax8-rtTA; LC1 mice; and Dr. Didier Auboeuf (INSERM, Lyon) for providing the *Ddx5* 3′UTR luciferase reporter plasmids. This work was supported by a grant from the 10.13039/501100001711Swiss National Science Foundation (SNSF) (grant no. 310030_2127121/1) to D.B.C., and in part using the resources and services of histology, imaging, and animal research core facilities at the School of Life Sciences of EPFL.

## Author contributions

Conceptualization: C.G., B.R., and D.B.C.; methodology, C.G., F.B., R.G., B.R., L.K., I.H., and D.B.C.; software, R.G. and B.R.; validation, C.G., B.R., and D.B.C.; formal analysis, C.G., B.R., and D.B.C.; investigation, C.G., F.B., R.C., and B.R.; resources, L.K. and I.H.; data curation, C.G., B.R., and D.B.C.; writing—original draft, C.G., B.R., and D.B.C.; review and editing, L.K., I.H., R.G., and A.B.; visualization, C.G., B.R., A.B., and D.B.C.; supervision, B.R. and D.B.C.; project administration, B.R. and D.B.C.; funding acquisition, D.B.C.

## Declaration of interests

The authors declare no competing interests.

## STAR★Methods

### Key resources table

**Table:** 

REAGENT or RESOURCE	SOURCE	IDENTIFIER
**Antibodies**		

Polyclonal anti-BICC1 antibody (rabbit)	Sigma	Cat# HPA045212; RRID: AB_10959667
Lotus tetragonolobus lectin (LTL)	Vector Labs	Cat# FL-1321; RRID: AB_2336559
DBA (Dolichos biflorus agglutinin, biotinylated)	Vector Labs	Cat# B-1035; RRID: AB_2314288
Monoclonal anti-phospho-CREB Ser133 (rabbit)	Cell Signaling	Cat# 9198; RRID: AB_2561044
Polyclonal anti-trimethyl-histone H3K27 (rabbit)	Millipore	Cat# 07–449; RRID: AB_310624
Polyclonal anti-DDX5 antibody (rabbit)	Bethyl	Cat# A300-523A; RRID: AB_451048
Polyclonal anti-YAP1 antibody (rabbit)	Proteintech	Cat# 13584-1-AP; RRID: AB_2218915
Monoclonal anti-γ-Tubulin (mouse)	Sigma	Cat# T6557; RRID: AB_477584
Anti-Mouse IgG (H + L) Highly Cross-Adsorbed Secondary Antibody, Alexa Fluor™ 568 (donkey)	Thermo Fisher Scientific	Cat# A-10037; RRID: AB_11180865
Streptavidin, Alexa Fluor® 647 conjugate	Thermo Fisher Scientific	Cat# S-21374; RRID: AB_2336066
IRDye® 680RD Donkey anti-Mouse IgG Secondary Antibody	LI-COR Biosciences	Cat# 926–68072; RRID: AB_2814912
IRDye® 800CW Donkey anti-Rabbit IgG Secondary Antibody	LI-COR Biosciences	Cat# 926–32213; RRID: AB_2715510

**Chemicals, peptides, and recombinant proteins**		

Doxycycline hyclate	Sigma	Cat# D9891
RNasin® Plus Ribonuclease Inhibitor	Promega	Cat# PRN2615
cOmplete™ Protease Inhibitor Cocktail	Roche	Cat# 11697498001
Phosphatase Inhibitor Cocktail 3	Sigma	Cat# P0044
jetPEI® DNA Transfection Reagent	Polyplus	Cat# 101-40N
Protein G Sepharose™ 4 Fast Flow	Cytiva	Cat# GE17-0618-01

**Critical commercial assays**		

Dual-Luciferase® Reporter Assay System	Promega	Cat# E1910
PrimeSTAR® Max DNA Polymerase	Takara	Cat# R045A
PowerUp™ SYBR™ Green Master Mix for qPCR	Applied Biosystems™	Cat# A25777
Phire Hot Start II DNA Polymerase (used for genotyping)	Thermo Fisher Scientific	Cat# F126

**Deposited data**		

ARTCyQ analysis pipeline code and trained model	This paper	Zenodo: https://doi.org/10.5281/zenodo.19066950
Bulk RNA-sequencing analysis pipeline	This paper	Zenodo: https://doi.org/10.5281/zenodo.21070463
Bulk RNA-sequencing data from Bicc1 cKO and control kidneys	This paper	GEO: GSE337067

**Experimental models: Cell lines**		

HEK293T	N/A	RRID: CVCL_0063
IMCD3-sh:ANKS3	Schlimpert et al.^83^	N/A

**Experimental models: Organisms/strains**		

C57BL/6JRj	The Jackson Laboratory	RRID: MGI:2670020
β-actin-Cre	The Jackson Laboratory	JAX stock #003376
Ksp1.3/Cre	The Jackson Laboratory	JAX stock #012237
Pax8-rtTA; TRE-LC1 mice	Dr. E. Hummler, UNIL	N/A
Bicc1^fl/fl^; Cdh16-Cre (cKO-Ksp)	This paper	N/A
Bicc1^fl/fl^; Pax8-rtTA; TRE-LC1	This paper	N/A

**Oligonucleotides**		

586-for TGCCGCCAAGAATATAACGAGA	This paper	N/A
587-rev TGGGTACCAGCTTGGAGTGT	This paper	N/A
588-for GCGTTTACACACGCAAGTGTGAG	This paper	N/A
549-rev CACAGGGCTCCTATGACCTC	This paper	N/A
*Anks3*-for TGTTAGCCAGGCAGTTTGGT	Rothé et al.^18^	N/A
*Anks3*-rev GAGGGACTTGCTCATAGGTGG	Rothé et al.^18^	N/A
*β-actin*-for ACAGAGCCTCGCCTTTGCC	Rothé et al.^18^	N/A
*β-actin*-rev CTCCATGCCCAGGAAGGAAGG	Rothé et al.^18^	N/A
*Ddx5*-for ATTTCCATCGGTCCTTGCCC	This paper	N/A
*Ddx5*-rev ATCTACCTCTTGTGCGGTGC	This paper	N/A

**Recombinant DNA**		

psiCHECK-2-Empty	Dardenne et al.^84^	N/A
psiCHECK-2-mDdx5-3′UTRprox	Dardenne et al.^84^	N/A

**Software and algorithms**		

QuPath 0.5.1 × 64	QuPath	RRID: SCR_018257
Fiji/ImageJ2, version 2.16.0/1.54p	ImageJ	RRID: SCR_002285
KNIME 5.3.2	KNIME	RRID: SCR_006164
OMERO	The Open Microscopy Environment	RRID: SCR_002629
STAR v2.7.10b	STAR	RRID: SCR_004463
DESeq2 v1.40.2	DESeq2	RRID: SCR_015687
g:Profiler	ELIXIR infrastructure	RRID: SCR_006809
EnrichR	Ma’ayan Lab	RRID: SCR_001575
Prism 10.4.0	GraphPad	RRID: SCR_002798

**Other**		

HM 325 microtome	Epredia	RRID: SCR_020259
CM1950 Cryostat	Leica	RRID: SCR_018061
VS200 Slide Scanner	Olympus	RRID: SCR_024783
Centro LB960 luminometer	Berthold	Cat# 38100-50
Mini-PROTEAN Tetra Vertical Electrophoresis Cell	Bio-Rad	Cat# 1658004
Trans-Blot Turbo Transfer System	Bio-Rad	Cat# 1704150
NovaSeq 6000 Sequencing System	Illumina	RRID: SCR_016387
Vevo 2100 Imaging Platform	FUJIFILM VisualSonics	RRID: SCR_015816
Odyssey CLx scanner	LI-COR Biosciences	RRID: SCR_014579

### Experimental model and study participant details

#### Generation of a conditional Bicc1 allele

*Bicc1*^em1Dbc^ mice (MGI:7485790) carrying a floxed *Bicc1* allele were generated by pronuclear microinjection in C57BL/6JRj zygotes. The injection mixture contained Cas9 protein (200 ng/μL, IDT), two sgRNAs (25 ng/μL each) targeting the intronic sequence flanking exon 4 of the *Bicc1* gene (5′-ACACTGATAAATGCGGATGT TGG-3′ and 5′-TACTTATGTAGCACTAACAAGGG-3′) and 15 ng/μL of a long single-stranded DNA (lssDNA) oligonucleotide containing exon 4 of *Bicc1* flanked by two *loxP* recombination sites and homology arms. The 3′ CRISPR target site contains a 49 bp insertion after the *loxP* site due to an unintended duplication of the lssDNA oligonucleotide.

#### Mouse strains and genotyping

All protocols for animal studies were in accordance with 3 R guidelines and with the animal welfare regulations of the Federal Food Safety and Veterinary Office of Switzerland and approved by the ethics committee and the veterinary administration of the canton of Vaud (license VD1722x6). Mice were maintained in individually ventilated cages at the EPFL animal facility. Routine PCR genotyping of wild-type and floxed *Bicc1* used the forward primer TGCCGCCAAGAATATAACGAGA (586) with the reverse primer TGGGTACCAGCTTGGAGTGT (587). A second PCR genotyping reaction used the forward primer GCGTTTACACACGCAAGTGTGAG (588) with the reverse primer CACAGGGCTCCTATGACCTC (549). Deletion of exon 4 by β-actin-Cre was verified using the primers 586 and 549. *Bicc1*^fl/fl^ mice were crossed with β-actin-Cre (JAX stock #003376), Ksp1.3/Cre (JAX stock #012237) or Pax8-rtTA; TRE-LC1 mice (provided by Dr. E. Hummler, UNIL). Genotyping of Ksp1.3/Cre and Pax8-rtTA; TRE-LC1 mice was performed as previously described.^34^^,^^85^ Pups were decapitated at designated endpoints, and adult mice were euthanized by CO_2_ inhalation. Kidneys were collected, weighed, and processed according to downstream applications. Deletion of *Bicc1* in cKO-P8LC1 mice was induced by administering doxycycline (200 mg/kg) in the chow for 10 days. Depending on the experiment, cKO-Ksp mice were analyzed at postnatal days P5, P7, P9, P11, or P14, whereas cKO-P8LC1 mice were analyzed at 7 months of age. The specific time points used are indicated in the corresponding figure legends. Both male and female mice were included in the study. Sex was considered as a biological variable in the kidney transcriptomic analysis. As genotype-associated transcriptional changes were largely shared between sexes, other experiments were not designed to assess sex-specific effects.

#### Cell lines

HEK293T cells (ATCC, CRL-11268) were purchased from ATCC. IMCD3-shANKS3 cells were provided by Dr. Gerd Walz.^83^ Both cell lines were cultured in DMEM (Sigma-Aldrich) supplemented with 10% fetal bovine serum (FBS; Sigma-Aldrich), 1% GlutaMAX (Thermo Fisher Scientific), and 1% gentamicin (Thermo Fisher Scientific) at 37°C in a humidified atmosphere containing 5% CO2.

### Method details

#### Quantitative analysis of cystic areas in kidney sections

Paraffin-embedded kidneys were sectioned at 4 μm and stained with hematoxylin and eosin (H&E). Whole-slide images were acquired using an Olympus slide scanner. Images were analyzed using QuPath and Fiji. For each kidney section, tissue regions and cystic or empty areas were delineated and quantified. Pixel classification and object detection were performed with QuPath in combination with custom Fiji macros, and measurements were exported as CSV files. Data were further processed and filtered using KNIME for downstream analysis.

#### Echography

Mice were anesthetized with isoflurane (1–3%) throughout the procedure (15–20 min) and placed on a heating platform in prone position. Cardiac function was monitored using four electrodes. Fur on the left and right flanks was removed with an electric shaver followed by a depilatory cream. Ultrasound gel was applied to facilitate transmission, and kidneys were imaged using a high-frequency ultrasound probe (30–40 MHz).

#### Histology and immunofluorescence staining

Kidneys were fixed in 4% paraformaldehyde (PFA) for variable durations depending on tissue size and washed three times in PBS. Samples were then processed either for paraffin embedding or for OCT embedding and cryosectioning. For paraffin processing, kidneys were embedded according to standard procedures, and sections were cut using an HM 325 microtome. Prior to staining, paraffin was removed using a standard automated dewaxing program, after which slides were immediately transferred to PBS to prevent drying. For OCT processing, following fixation and PBS washes, PBS was replaced with 15% sucrose until tissues sank (2–24 h), then embedded in OCT block and snap-frozen in an isopentane bath cooled with dry ice. Once frozen, blocks were stored at −80°C. Before sectioning, blocks were equilibrated to −20°C and sectioned using a CM1950 cryostat. Sections were stored at −80°C and brought to room temperature prior to staining. Sections were permeabilized for 10 min in PBS containing 0.25% Triton X-100. For immunofluorescent staining, sections were blocked with 1% BSA in PBS for 2 h at room temperature prior to incubation with the primary antibody. For biotinylated probes, an additional blocking step was performed using a biotin-streptavidin blocking kit according to the manufacturer’s instructions (Vector Laboratories, SP-2002). Primary antibodies were diluted in PBS containing 1% BSA and incubated overnight at 4°C under gentle agitation. Sections were washed three times in PBS at room temperature for 5 min each. Alexa Fluor-conjugated secondary antibodies or streptavidin diluted in PBS containing 1% BSA were incubated for 45 min at room temperature under agitation, followed by three PBS washes. After nuclear counterstaining with DAPI (1:10,000) for 10 min at room temperature, sections were rinsed three times in PBS and mounted using Fluoromount-G mounting medium. Imaging was performed at 40× on Olympus VS200 slide scanner.

#### Luciferase assays

Luciferase assays were carried out using the Dual-Luciferase Reporter Assay System (Promega), as previously described.^84^ Briefly, 100,000 HEK293T cells were plated in 24-well plates. After 12 h, triplicate samples were transfected with the dual-luciferase plasmids psiCHECK-2-Empty or psiCHECK-2-mDdx5-3′UTRprox (0.1 μg/well) using jetPEI (Polyplus Transfection). Renilla luciferase was used as the reporter, while Firefly luciferase served as an internal normalization control. Firefly and Renilla luciferase activities were measured using a Centro LB960 luminometer (Berthold).

#### Western blotting

Western blot analysis was performed by standard procedures using a fluorescence-based detection method according to the manufacturer’s recommendations for the Odyssey CLx imaging system. Fluorescent signals were acquired and quantified using Odyssey CLx scanner software, ensuring that all bands were within the linear dynamic range and below saturation.

#### RNA co-immunoprecipitation assay in IMCD3 cells

IMCD3-sh*Anks3* cells were seeded at a density of 2 × 10^6^ per 10 cm dish in medium containing 0.25 μg/mL doxycycline. After 2 days, cells were passaged into two 15 cm dishes at a density of 5 × 10^6^ cells per plate and doxycycline was added in fresh complete medium every 48 h for 5 days. RNA co-immunoprecipitation was performed as previously described.^18^ RT-qPCR was performed using the following oligonucleotide primers: ATTTCCATCGGTCCTTGCCC and ATCTACCTCTTGTGCGGTGC for *Ddx5* mRNA, TGTTAGCCAGGCAGTTTGGT and GAGGGACTTGCTCATAGGTGG for *Anks3* mRNA, or ACAGAGCCTCGCCTTTGCC and CTCCATGCCCAGGAAGGAAGG for *β-actin* mRNA. The ratio of bound/total mRNA was calculated with the formula 2^−Ct(IP)^/2^−Ct(Input)^. To account for fluctuations between samples in the amounts of immunoprecipitated Bicc1, the resulting values were normalized to the intensity of the Bicc1 protein signal measured by Western blot in immunoprecipitates, and *Ddx5* mRNA enrichment was reported relative to that of *β-actin* mRNA as an internal reference for non-specific RNA binding. Finally, to visualize the effect of the doxycycline-induced Anks3 depletion, results were expressed relative to the condition without doxycycline.

#### Automated renal tubule and cyst quantification (ARTCyQ)

Whole kidney sections were stained for immunofluorescence and imaged on five channels (DAPI, FITC, Cy3, Cy5, and Cy7). Tubules in control kidneys were segmented and classified using the deep neural network Omnipose^36^ trained on sections that were fluorescently labeled with nephron segment-specific markers. Cysts in *Bicc1* mutant kidneys were detected using DAPI-based pixel classifiers and classified based on marker staining in surrounding epithelial cells. Segmented objects were subdivided into cytoplasmic and nuclear compartments, and morphological features and fluorescence intensities were extracted for each object. Each object was assigned a unique identifier to maintain traceability between the original images and the processed data.

#### Bulk RNA-seq analysis

Mouse RNA samples were pooled, and Illumina Stranded mRNA Ligation (ISML) libraries were prepared. Libraries were sequenced on the NovaSeq 6000 platform using paired-end 60 bp (PE60) reads, yielding approximately 60 million reads per sample. Reads were then trimmed to remove ISML (Nextera) adapter sequences.

FASTQ reads were aligned to the mouse mm10 reference genome using STAR (v2.7.10b),^86^ and gene-level counts were generated based on annotations from Ensembl release 102. Genes were retained for downstream analysis if they had more than 10 counts in at least 4 of the 16 samples, resulting in 18,865 expressed genes. Variance-stabilizing transformation was applied for principal component analysis (PCA) and sample clustering. Based on the PCA results, there was no need for batch correction. Differential expression analysis was performed separately for each sex using default settings in DESeq2 (v1.40.2).^87^ A complementary DESeq2 analysis was performed on the full dataset using sex as a covariate in the design formula (∼ sex + genotype). Genes were considered differentially expressed at an adjusted *p*-value ≤0.05. Over-representation analysis was conducted using the g:Profiler R client^88^ to identify enriched GO and KEGG pathways. Expressed genes were used as the custom background (domain scope), and enrichment was assessed separately for upregulated and downregulated gene sets.

To assess overlap between the differentially expressed genes (DEGs) in the *Bicc1* cKO-Ksp samples and the meta-analysis reported by Malas et al. (2017), we first identified genes that were consistently up- or downregulated in the same direction in both males and females in our dataset. These genes were then compared with the gene lists *Data1_DEGs.xlsx* (PKD signature), *Data2_meta.xlsx* (PKD meta-analysis), and *Data4_InjuryRepairProfiles.xlsx* (PKD injury-repair signatures) of Malas et al.^40^, where the directionality of gene expression changes in *Data2_meta.xlsx* matches the one in *Data1_DEGs.xlsx* in most instances. Gene matching was performed using either the *entrez_id* (when available) or the associated gene name and overlaps between the gene sets were visualized. In rare instances, the directionality of gene expression changes reported in the Malas meta-analysis and its *Data1_DEGs.xlsx* file did not match.

#### Gene set enrichment analysis

A consensus transcription factor (TF)–target gene library was generated by integrating multiple publicly available ChIP-seq–based datasets from EnrichR, including ENCODE TF ChIP-seq (2015), ChEA (2022), and the ENCODE and ChEA Consensus TFs from ChIP-X. The datasets were merged, curated, and standardized using a KNIME workflow to produce a non-redundant reference of TF-target associations. This library was then cross-referenced with differentially expressed gene (DEG) lists from the experimental conditions to identify candidate TFs regulating the observed transcriptional changes. Enrichment of target genes of each TF within each DEG set was assessed relative to the defined gene universe using Fisher’s exact test. The number of DEGs versus non-DEGs that are bound by each TF, and the number of those that are not, were calculated to derive 2x2 contingency counts. Odds ratios (OR) with 95% confidence intervals (CI) were computed. One-tailed tests were used to detect enrichment, and *p*-values were adjusted for multiple testing using the Benjamini-Hochberg procedure to control the false discovery rate (FDR). For visualization, log_2_(OR) was plotted against -log_10_(FDR), with FDR values equal to zero set to the smallest non-zero FDR observed. To preserve relative significance and directionality, and to avoid generating infinite values during log-transformation, OR values equal to zero were set to the smallest non-zero OR observed. Analyses were performed using Excel and GraphPad Prism.

### Quantification and statistical analysis

Data are presented as mean ± standard deviation (SD) unless otherwise indicated. Comparisons between two groups were performed using Student’s *t* test for normally distributed data. For comparisons involving more than two groups or non-normally distributed data, the Kruskal-Wallis test followed by Dunn’s post-hoc test for multiple comparisons was applied. Enrichment of transcription factor (TF) target genes within DEG sets was assessed using Fisher’s exact test, and *p*-values were adjusted for multiple testing using the Benjamini-Hochberg procedure to control the false discovery rate (FDR). *p*-values <0.05 were considered statistically significant. All analyses were performed using Excel and GraphPad Prism.

## Supplemental information

Document S1. Figures S1–S8

Table S1. Nuclear immunofluorescence intensities of Bicc1 targets quantified by ARTCyQ, related to Figure 3

Table S2. Cyst segment classification and lumenal area analysis by ARTCyQ, related to Figure 4

Table S3. RNA-seq analysis, related to Figure 5

Table S4. KEGG GO terms, related to Figure 5

Table S5. Meta-analysis, related to Figure 5

Table S6. Unified Enrichr ChIP-seq transcription factor binding site library, related to Figure 6

Table S7. GSEA of PKD signature and related Bicc1 target DEGs using a unified EnrichR transcription factor binding sites library, related to Figure 6

Table S8. OR and FDR values for transcription factor binding site enrichment analyses, related to Figure 6

Table S9. Reporting of individual data points and statistical analysis, related to Figure S4
