# Supplementary material for: Kidney-specific deletion of the BicC family RNA-binding protein 1 triggers an ADPKD-like cystogenic program
Source: iScience. 2026 Aug 3;29(8):117020. doi: 10.1016/j.isci.2026.117020 (PMC13469810; doi:10.1016/j.isci.2026.117020)
Supplement: Supplementary file 1 [file mmc1.pdf]

iScience, Volume *29*

## Supplemental information

# Kidney-specific deletion of the BicC family RNA-binding protein 1 triggers an ADPKD-like cystogenic program

**Céline Gagnieux, Florian Bernet, Lona Kroese, Romain Guiet, Allison Burns, Roy Combe, Ivo Huijbers, Daniel B. Constam, and Benjamin Rothé**

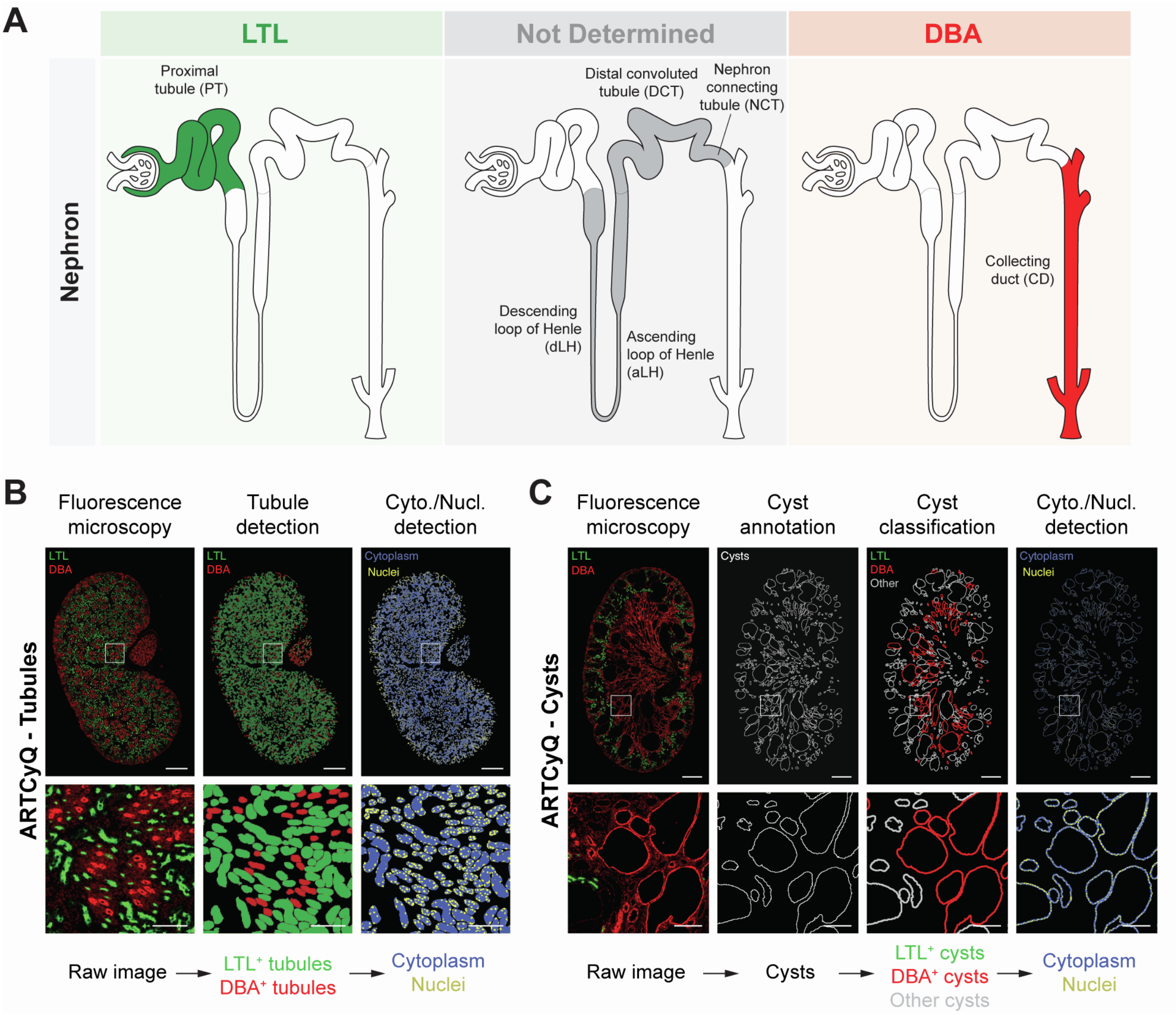


**Figure S1. Automated Renal Tubule and Cyst Quantification (ARTCyQ) workflow.**
(A) ARTCyQ pipeline for renal tubule identification. Proximal tubules ($LTL^+$) and collecting ducts ($DBA^+$) were detected using a trained Omnipose model. Each detected tubule was subsequently segmented into cytoplasmic and nuclear compartments using a pixel classifier. (B) ARTCyQ pipeline for renal cyst identification. Cyst-lining cells were first detected based on lumen morphology using a pixel classifier, then classified according to lectin marker signal (LTL or DBA), and segmented into cytoplasmic and nuclear compartments. Cysts lacking detectable LTL or DBA signal were classified as “not determined” (ND). (C) Overview of object classes generated by ARTCyQ. The Tubule class comprises marker-defined structures identified in control kidneys, including $LTL^+$ PTs and $DBA^+$ CDs. The Cyst class comprises cyst-lining cells identified in cKO-Ksp kidneys and includes $LTL^+$, $DBA^+$, and “ND” subclasses. Note that the “ND” category is absent from the Tubule class because tubule identification requires marker expression, whereas cyst identification is initiated by lumen detection independently of segment markers. In (A) and (B), scale bars: 500 μm (whole kidney sections, top panels) and 100 μm (magnified views, bottom panels).

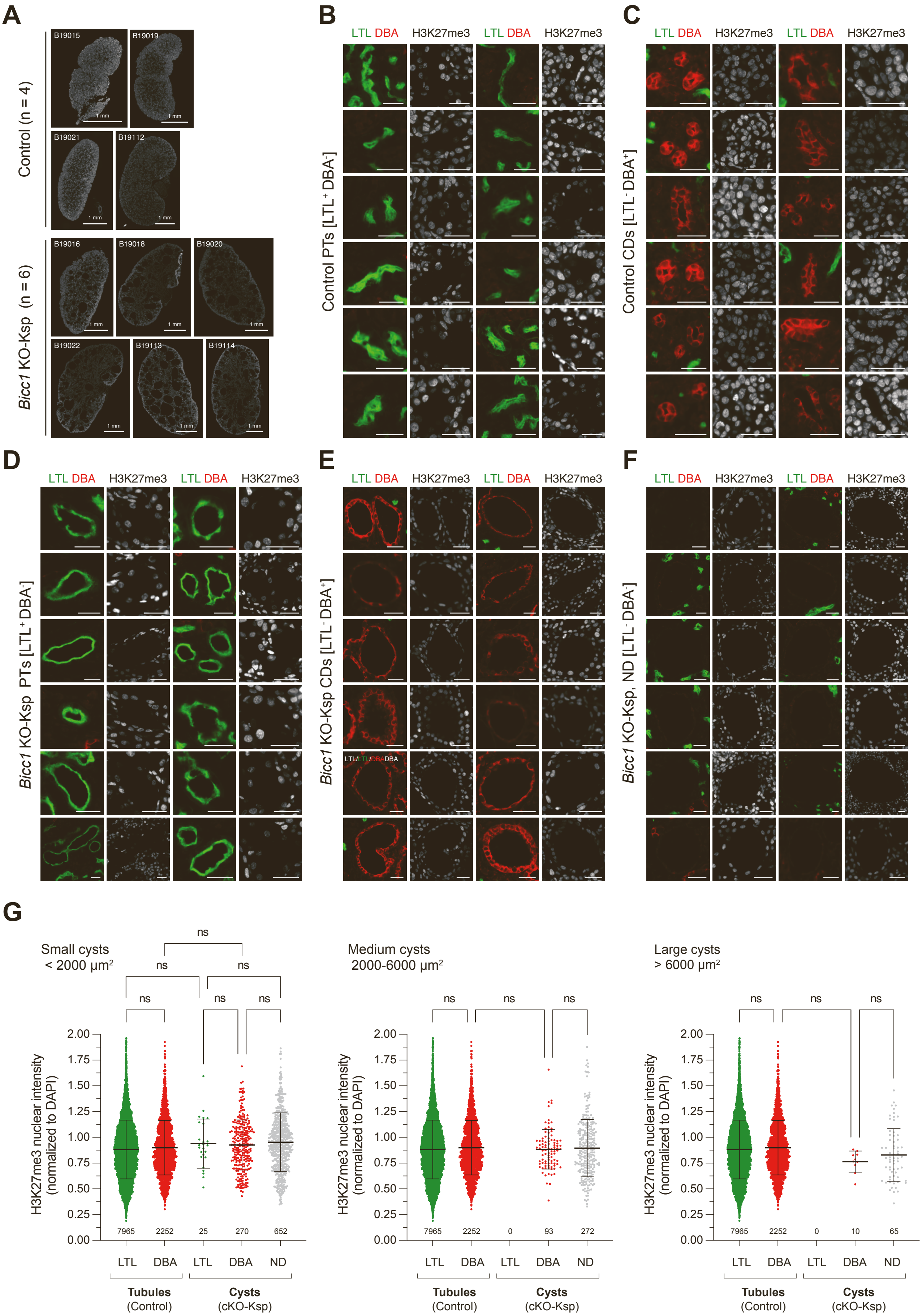

A
Control (n = 4)
B19015
B19019
B19021
B19112
Bicc1 KO-Ksp (n = 6)
B19016
B19018
B19020
B19022
B19113
B19114
1 mm
B
LTL DBA
H3K27me3
Control PTs [LTL⁺ DBA⁻]
C
Control CDs [LTL⁻ DBA⁺]
D
Bicc1 KO-Ksp PTs [LTL⁺ DBA⁻]
E
Bicc1 KO-Ksp CDs [LTL⁻ DBA⁺]
F
Bicc1 KO-Ksp, ND [LTL⁻ DBA⁻]
G
Small cysts
< 2000 µm²
Medium cysts
2000-6000 µm²
Large cysts
> 6000 µm²
ns
H3K27me3 nuclear intensity (normalized to DAPI)
0.00
0.25
0.50
0.75
1.00
1.25
1.50
1.75
2.00
7965
2252
25
270
652
93
272
0
10
65
LTL
DBA
ND
Tubules (Control)
Cysts (cKO-Ksp)

**Figure S2. ARTCyQ analysis of nuclear H3K27me3 levels in *Bicc1* cKO-Ksp kidneys.**

(A) DAPI staining of individual kidney sections used for quantitative analysis in figure 3A. (B-F) Representative immunofluorescence images of H3K27me3 (white) in kidney sections from *Bicc1* cKO-Ksp mice and control littermates at postnatal day P5. Sections were counterstained with LTL (green) and DBA (red) to label PTs and CDs, respectively. (G) Quantification of nuclear H3K27me3 fluorescence intensity from the dataset in figure 3A, stratified into three classes according to cyst size: small (< 2000 $\mu m^2$), medium (2000-6000 $\mu m^2$), and large (> 6000 $\mu m^2$). Nuclear H3K27me3 intensity was normalized to DAPI signal for each object. Each data point represents an individual tubule or cyst, with signal intensities averaged across all nuclei within that object. n indicates the number of analyzed objects. Statistical analysis was performed using the Kruskal-Wallis test followed by Dunn's post-hoc test for multiple comparisons. Data are presented as mean ± standard deviation (SD) from 4 control and 6 cKO-Ksp animals. Exact p-values are indicated in the graphs; ns, not significant.

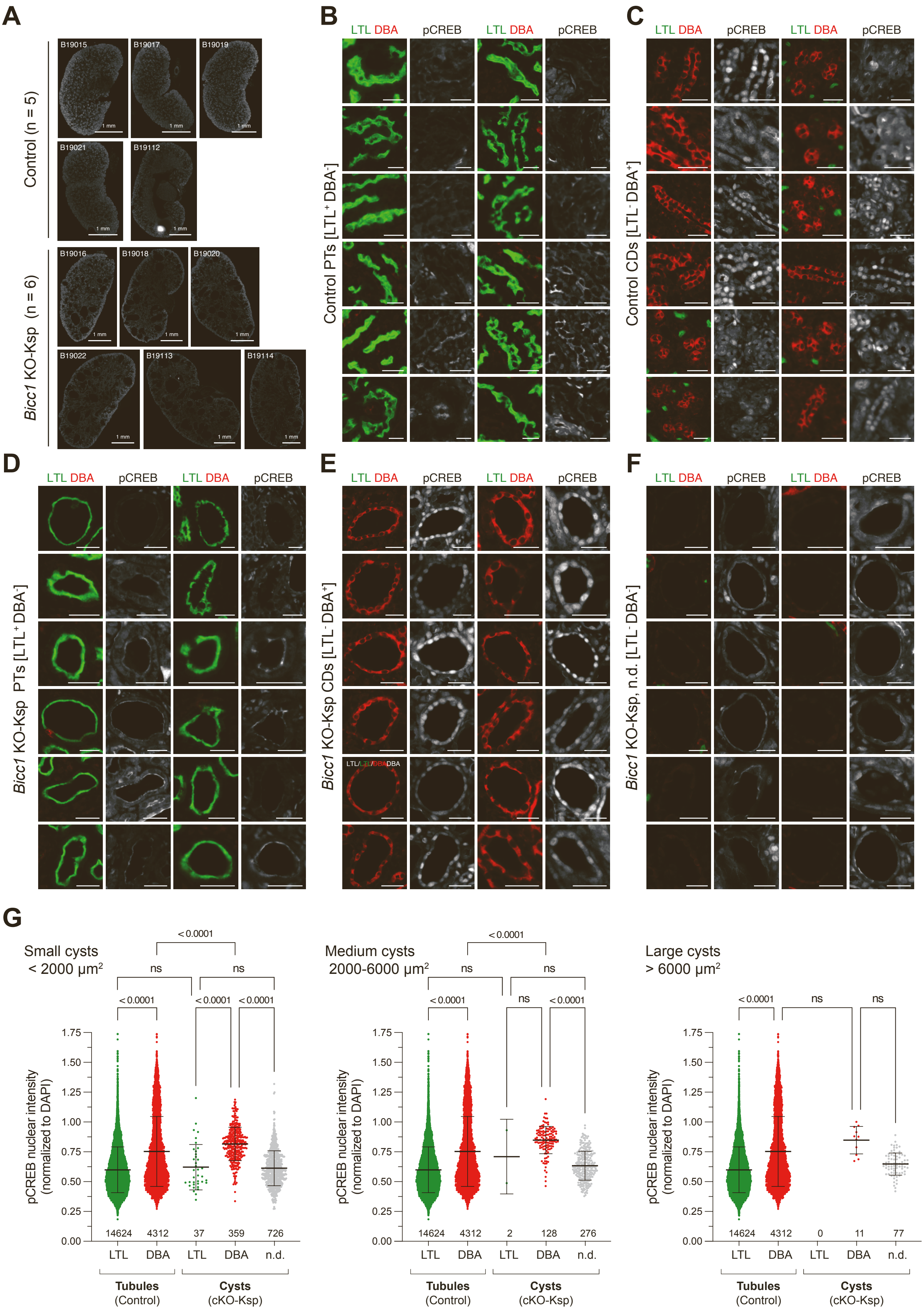
A
Control (n = 5)
B19015
B19017
B19019
B19021
B19112
1 mm
Bicc1 KO-Ksp (n = 6)
B19016
B19018
B19020
B19022
B19113
B19114
B
LTL DBA
pCREB
Control PTs [LTL+ DBA-]
C
Control CDs [LTL- DBA+]
D
Bicc1 KO-Ksp PTs [LTL+ DBA-]
E
Bicc1 KO-Ksp CDs [LTL- DBA+]
LTL/LTL/DBA/DBA
F
Bicc1 KO-Ksp, n.d. [LTL- DBA-]
G
Small cysts
< 2000 µm2
Medium cysts
2000-6000 µm2
Large cysts
> 6000 µm2
pCREB nuclear intensity
(normalized to DAPI)
< 0.0001
ns
0.00
0.25
0.50
0.75
1.00
1.25
1.50
1.75
14624
4312
37
359
726
2
128
276
0
11
77
LTL
DBA
n.d.
Tubules
(Control)
Cysts
(cKO-Ksp)

**Figure S3. ARTCyQ analysis of nuclear pCREB levels in Bicc1 cKO-Ksp kidneys.**

(A) DAPI staining of individual kidney sections used for quantitative analysis in Figure 3B. (B-F) Representative immunofluorescence images of pCREB (white) in kidney sections from postnatal day 5 (P5) Bicc1 cKO-Ksp mice and control littermates. Sections were counterstained with LTL (green) and DBA (red). (G) Quantification of nuclear pCREB fluorescence intensity from the dataset shown in figure 3B, stratified into three classes according to cyst size: small (< 2000 $\mu m^2$), medium (2000-6000 $\mu m^2$), and large (> 6000 $\mu m^2$). Nuclear pCREB intensity was normalized to DAPI signal for each object. Each data point represents an individual tubule or cyst, with signal intensities averaged across all nuclei within that object. n indicates the number of analyzed objects. Statistical analysis was performed using the Kruskal-Wallis test followed by Dunn's post-hoc test for multiple comparisons. Data are presented as mean ± standard deviation (SD) from 5 control and 6 cKO-Ksp animals. Exact p-values are indicated in the graphs; ns, not significant.

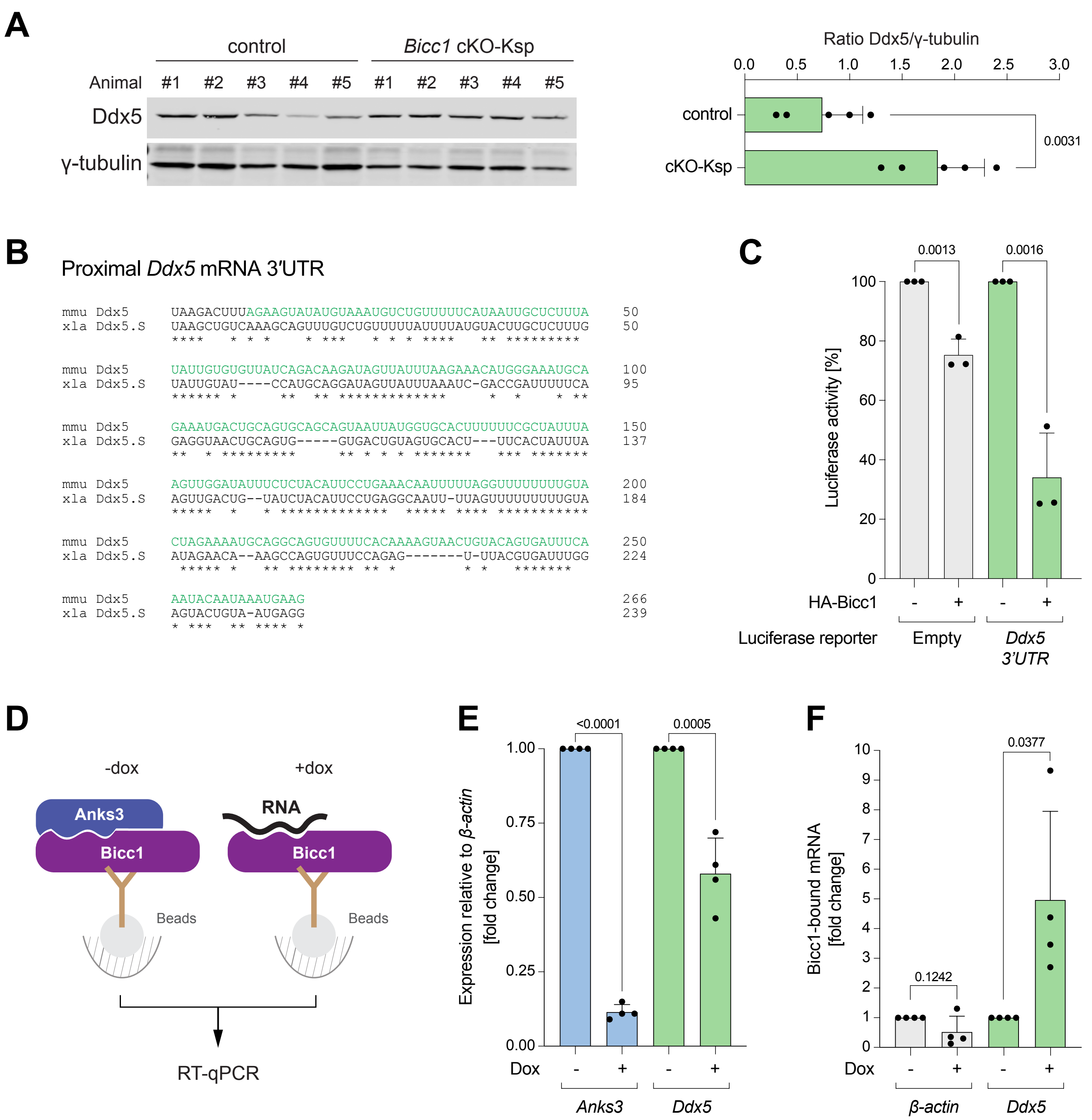
A
control
Bicc1 cKO-Ksp
Animal
#1 #2 #3 #4 #5 #1 #2 #3 #4 #5
Ddx5
γ-tubulin
Ratio Ddx5/γ-tubulin
0.0 0.5 1.0 1.5 2.0 2.5 3.0
control
cKO-Ksp
0.0031
B
Proximal Ddx5 mRNA 3′UTR
mmu Ddx5 UAAGACUUUAGAAGUAUAUGUAAAUGUCUGUUUUUCAUAAUUGCUCUUUA 50
xla Ddx5.S UAAGCUGUCAAAGCAGUUUGUCUGUUUUUAUUUUAUGUACUUGCUCUUUG 50
mmu Ddx5 UAUUGUGUGUUAUCAGACAAGAUAGUUAUUUAAGAAACAUGGGAAAUGCA 100
xla Ddx5.S UAUUGUAU----CCAUGCAGGAUAGUUAUUUAAAUC-GACCGAUUUUUCA 95
mmu Ddx5 GAAAUGACUGCAGUGCAGCAGUAAUUAUGGUGCACUUUUUUCGCUAUUUA 150
xla Ddx5.S GAGGUAACUGCAGUG-----GUGACUGUAGUGCACU---UUCACUAUUUA 137
mmu Ddx5 AGUUGGAUAUUUCUCUACAUUCCUGAAACAAUUUUUAGGUUUUUUUUGUA 200
xla Ddx5.S AGUUGACUG--UAUCUACAUUCCUGAGGCAAUU-UUAGUUUUUUUUUGUA 184
mmu Ddx5 CUAGAAAAUGCAGGCAGUGUUUUCACAAAAGUAACUGUACAGUGAUUUCA 250
xla Ddx5.S AUAGAACA--AAGCCAGUGUUUCCAGAG-------U-UUACGUGAUUUGG 224
mmu Ddx5 AAUACAAUAAAUGAAG 266
xla Ddx5.S AGUACUGUA-AUGAGG 239
C
0.0013
0.0016
Luciferase activity [%]
0 20 40 60 80 100
HA-Bicc1
- + - +
Luciferase reporter
Empty
Ddx5 3′UTR
D
-dox
+dox
Anks3
Bicc1
RNA
Bicc1
Beads
Beads
RT-qPCR
E
<0.0001
0.0005
Expression relative to β-actin [fold change]
0.00 0.25 0.50 0.75 1.00
Dox
- + - +
Anks3
Ddx5
F
0.1242
0.0377
Bicc1-bound mRNA [fold change]
0 1 2 3 4 5 6 7 8 9 10
Dox
- + - +
β-actin
Ddx5

**Figure S4. Bicc1 associates with *Ddx5* mRNA and represses its expression through the 3′UTR.**

(A) Western blot analysis and quantification of Ddx5 protein levels in control and cKO-Ksp kidneys at P9. Ddx5 levels were normalized to γ-tubulin and expressed relative to control kidneys. (B) Pairwise alignment of the proximal *Ddx5* mRNA 3′UTR between mouse and Xenopus, with the mouse 3′UTR sequence used in the luciferase reporter highlighted in green. (C) Luciferase reporter assay in HEK293T cells transfected with an empty reporter or a reporter carrying the mouse *Ddx5* 3′UTR, in the absence or presence of HA-Bicc1. Luciferase activity is expressed relative to the mock-transfected condition. (D) Cartoon depicting the experimental conditions of RNA co-immunoprecipitation by Bicc1 in doxycycline (Dox)-inducible sh::*Anks3* IMCD3 cells. (E) RT-qPCR analysis of endogenous *Anks3* and *Ddx5* mRNAs in IMCD3 cells treated with or without 0.25 µg/ml doxycycline to induce the expression of *Anks3* shRNA. The levels of transcripts relative to *β-actin* mRNAs is expressed as a percentage of the baseline in untreated cells. (F) RT-qPCR analysis of *Ddx5* and *β-actin* mRNAs in IMCD3 before (-dox) and after (+dox) *Anks3* depletion in IMCD3 cells. The y-axis shows the bound/total mRNA ratio in Bicc1 immunoprecipitates after normalization to Bicc1 protein levels in the IP fraction, expressed as fold change relative to the control condition (-dox). Data are means + SD from independent experiments or biological replicates, as indicated. Statistical significance was assessed by Student's t test. Exact p-values are indicated in the graphs; ns, not significant. Underlying data can be found in the Supplementary Table S9.

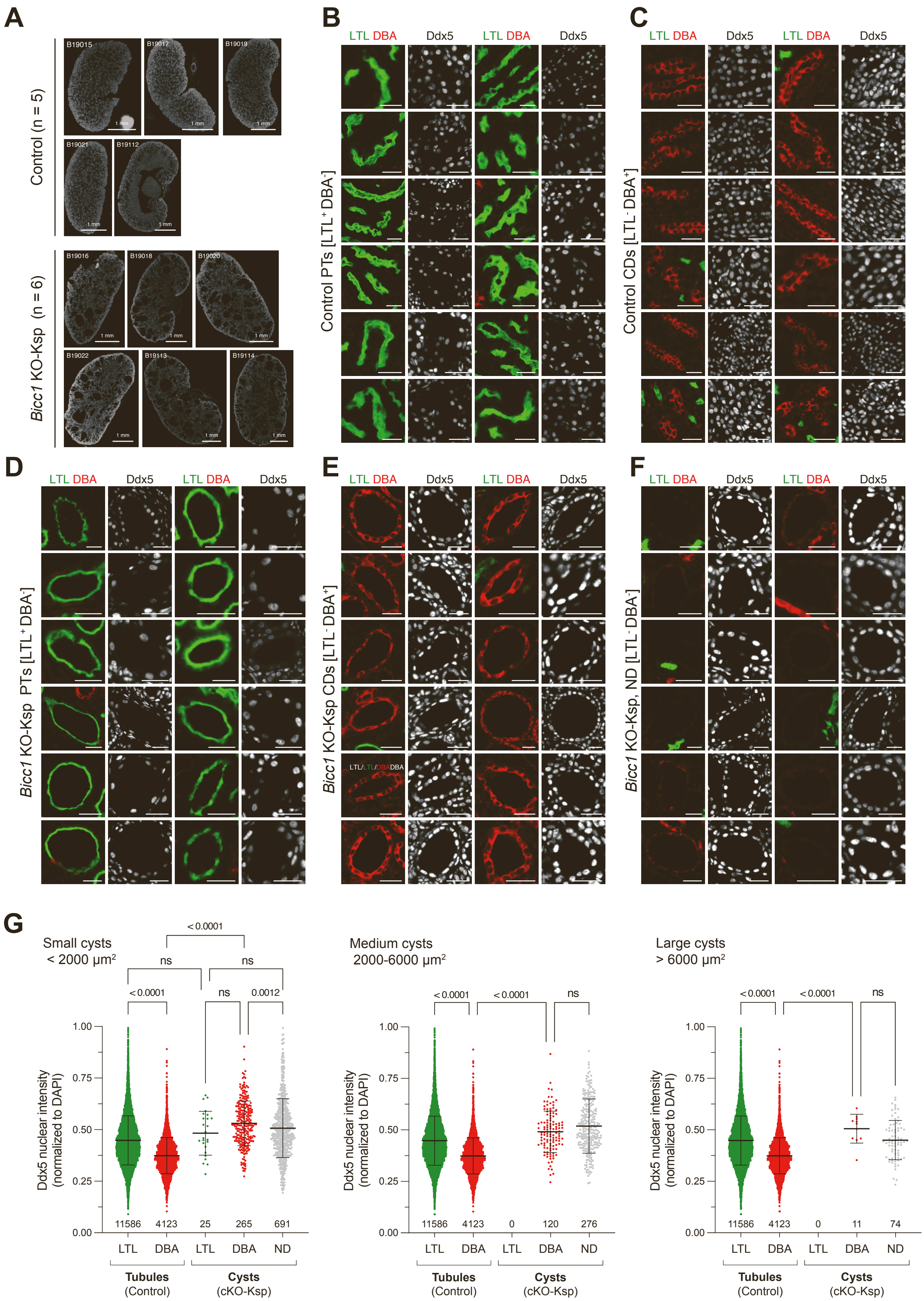
A
Control (n = 5)
B19015
B19017
B19019
B19021
B19112
1 mm
Bicc1 KO-Ksp (n = 6)
B19016
B19018
B19020
B19022
B19113
B19114
B
C
D
E
F
LTL DBA
Ddx5
Control PTs [LTL+ DBA-]
Control CDs [LTL- DBA+]
Bicc1 KO-Ksp PTs [LTL+ DBA-]
Bicc1 KO-Ksp CDs [LTL- DBA+]
Bicc1 KO-Ksp, ND [LTL- DBA-]
G
Small cysts
< 2000 µm²
Medium cysts
2000-6000 µm²
Large cysts
> 6000 µm²
< 0.0001
ns
0.0012
Ddx5 nuclear intensity
(normalized to DAPI)
1.00
0.75
0.50
0.25
0.00
11586
4123
25
265
691
0
120
276
11
74
LTL
DBA
ND
Tubules
(Control)
Cysts
(cKO-Ksp)

**Figure S5. ARTCyQ analysis of nuclear Ddx5 levels in *Bicc1* cKO-Ksp kidneys.**

(A) DAPI staining of individual kidney sections used for quantitative analysis in figure 3C. (B-F) Representative immunofluorescence images of Ddx5 (white) in kidney sections from *Bicc1* cKO-Ksp mice and control littermates at postnatal day P5. Sections were counterstained with LTL (green) and DBA (red). (G) Quantification of nuclear Ddx5 fluorescence intensity from the dataset in figure 3C, stratified into three classes according to cyst size: small (< 2000 $\mu m^2$), medium (2000-6000 $\mu m^2$), and large (> 6000 $\mu m^2$). Nuclear Ddx5 intensity was normalized to DAPI signal for each object. Each data point represents an individual tubule or cyst, with signal intensities averaged across all nuclei within that object. n indicates the number of analyzed objects. Statistical analysis was performed using the Kruskal-Wallis test followed by Dunn's post-hoc test for multiple comparisons. Data are presented as mean ± standard deviation (SD) from 5 control and 6 cKO-Ksp animals. Exact p-values are indicated in the graphs; ns, not significant.

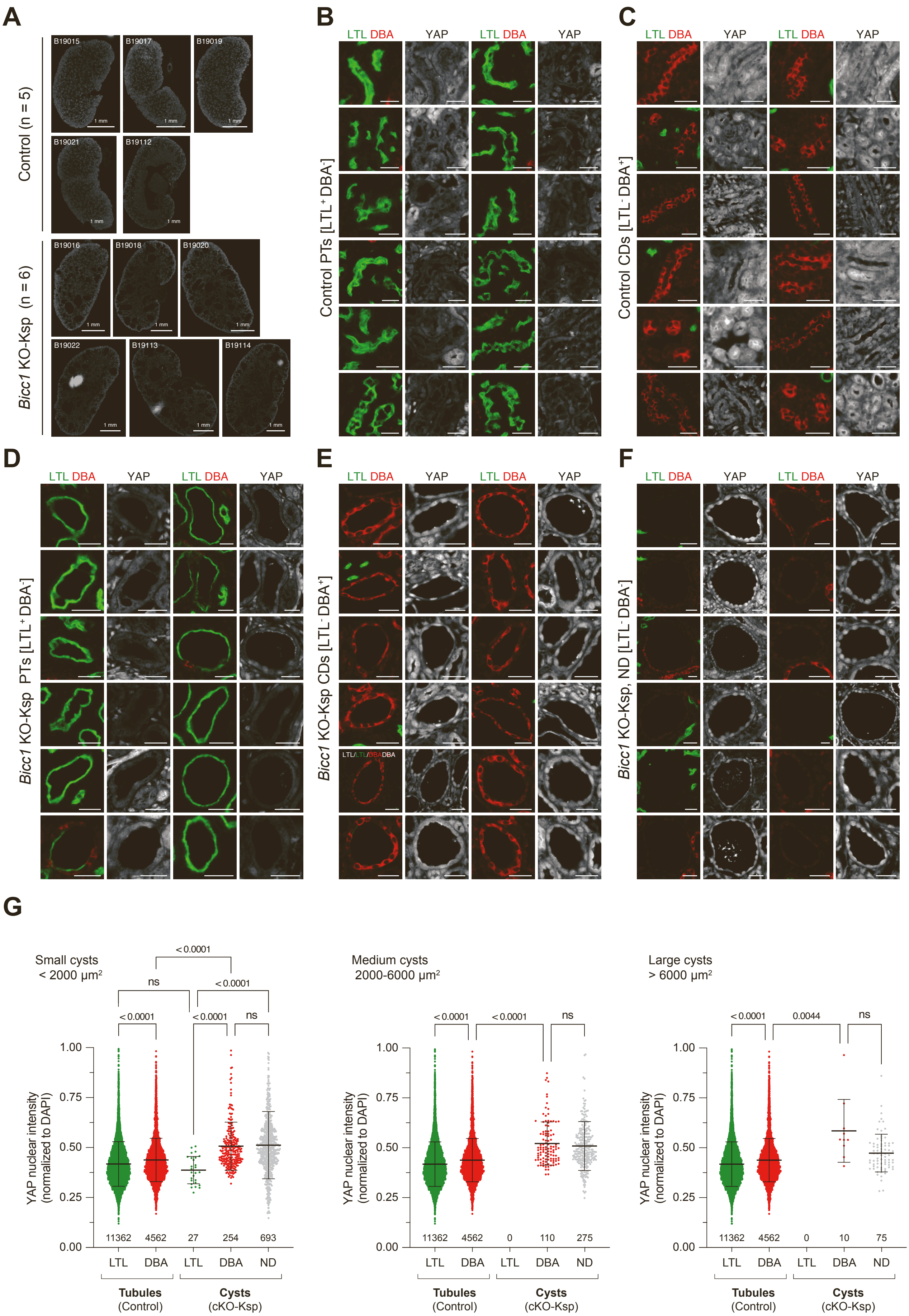
A
Control (n = 5)
Bicc1 KO-Ksp (n = 6)
B19015
B19017
B19019
B19021
B19112
B19016
B19018
B19020
B19022
B19113
B19114
1 mm
B
Control PTs [LTL+ DBA-]
C
Control CDs [LTL- DBA+]
D
Bicc1 KO-Ksp PTs [LTL+ DBA-]
E
Bicc1 KO-Ksp CDs [LTL- DBA+]
F
Bicc1 KO-Ksp, ND [LTL- DBA-]
LTL DBA
YAP
G
Small cysts
< 2000 µm²
Medium cysts
2000-6000 µm²
Large cysts
> 6000 µm²
YAP nuclear intensity
(normalized to DAPI)
1.00
0.75
0.50
0.25
0.00
< 0.0001
ns
0.0044
11362
4562
27
254
693
0
110
275
10
75
LTL
DBA
ND
Tubules
(Control)
Cysts
(cKO-Ksp)

**Figure S6. ARTCyQ analysis of nuclear YAP levels in Bicc1 cKO-Ksp kidneys.**

(A) DAPI staining of individual kidney sections used for quantitative analysis in Figure 3D. (B-F) Representative immunofluorescence images of YAP (white) in kidney sections from *Bicc1* cKO-Ksp mice and control littermates at postnatal day P5. Sections were counterstained with LTL (green) and DBA (red). (G) Quantification of nuclear YAP fluorescence intensity from the dataset in figure 3D, stratified into three classes according to cyst size: small (< 2000 μm$^2$), medium (2000-6000 μm$^2$), and large (> 6000 μm$^2$). Nuclear pCREB intensity was normalized to DAPI signal for each object. Each data point represents an individual tubule or cyst, with signal intensities averaged across all nuclei within that object. n indicates the number of analyzed objects. Statistical analysis was performed using the Kruskal-Wallis test followed by Dunn's post-hoc test for multiple comparisons. Data are presented as mean ± standard deviation (SD) from 5 control and 6 cKO-Ksp animals. Exact p-values are indicated in the graphs; ns, not significant.

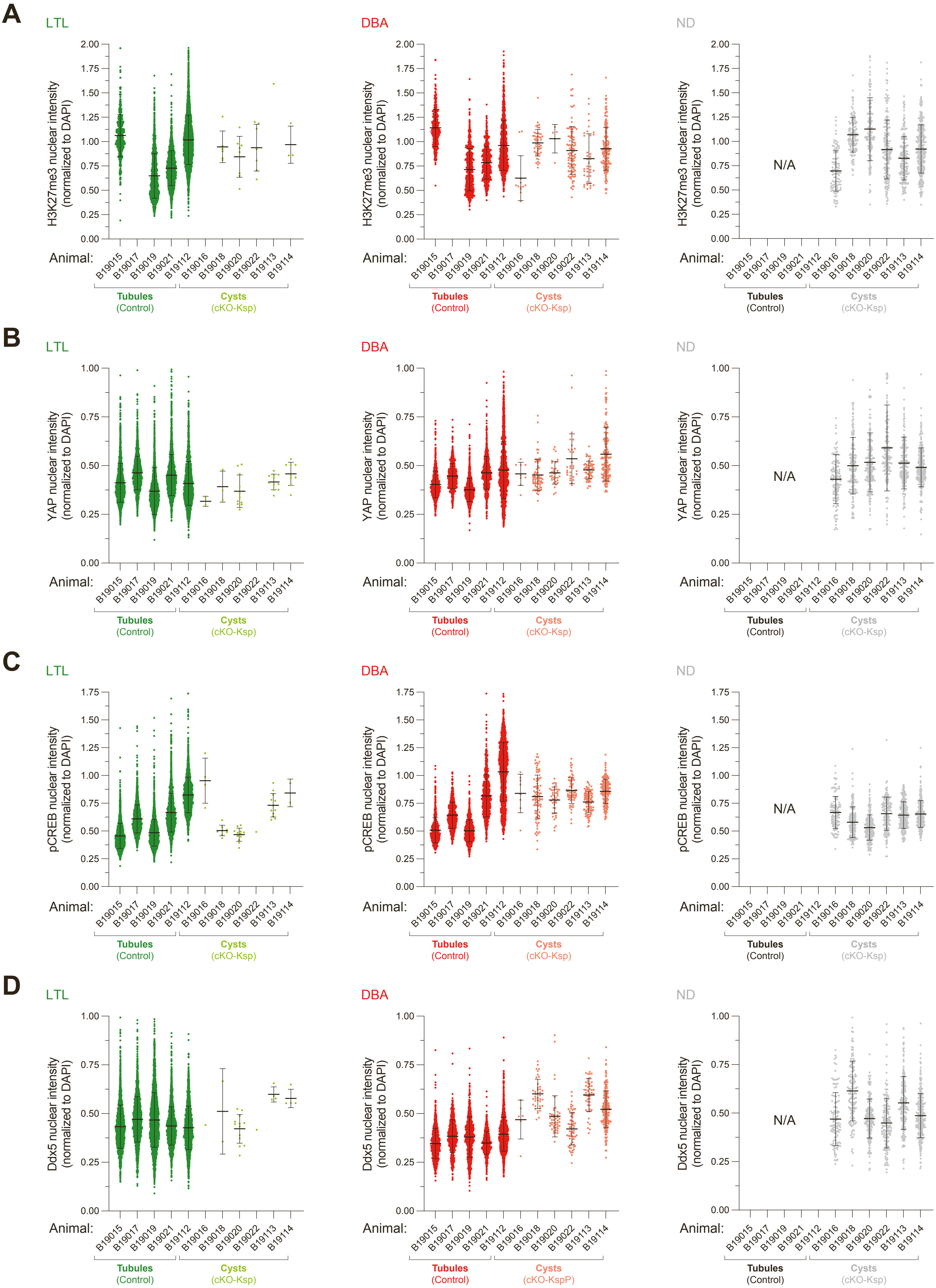
A
LTL
DBA
ND
H3K27me3 nuclear intensity (normalized to DAPI)
2.00
1.75
1.50
1.25
1.00
0.75
0.50
0.25
0.00
Animal:
B19015
B19017
B19019
B19021
B19112
B19016
B19018
B19020
B19022
B19113
B19114
Tubules (Control)
Cysts (cKO-Ksp)
N/A
B
YAP nuclear intensity (normalized to DAPI)
1.00
0.75
0.50
0.25
0.00
C
pCREB nuclear intensity (normalized to DAPI)
1.75
1.50
1.25
1.00
0.75
0.50
0.25
0.00
D
Ddx5 nuclear intensity (normalized to DAPI)
1.00
0.75
0.50
0.25
0.00
Cysts (cKO-KspP)

**Figure S7.** ARTCyQ measurements plotted per individual animal.

ARTCyQ quantifications of nuclear H3K27mme3 (A), pCREB (B), Ddx5 (C) and YAP (D) levels displayed separately for each control and *Bicc1* cKO-Ksp. Data points represent individual tubules or cysts, classified and quantified as described in Figure 3. Horizontal bars and error bars indicate the mean ± SD for each animal.

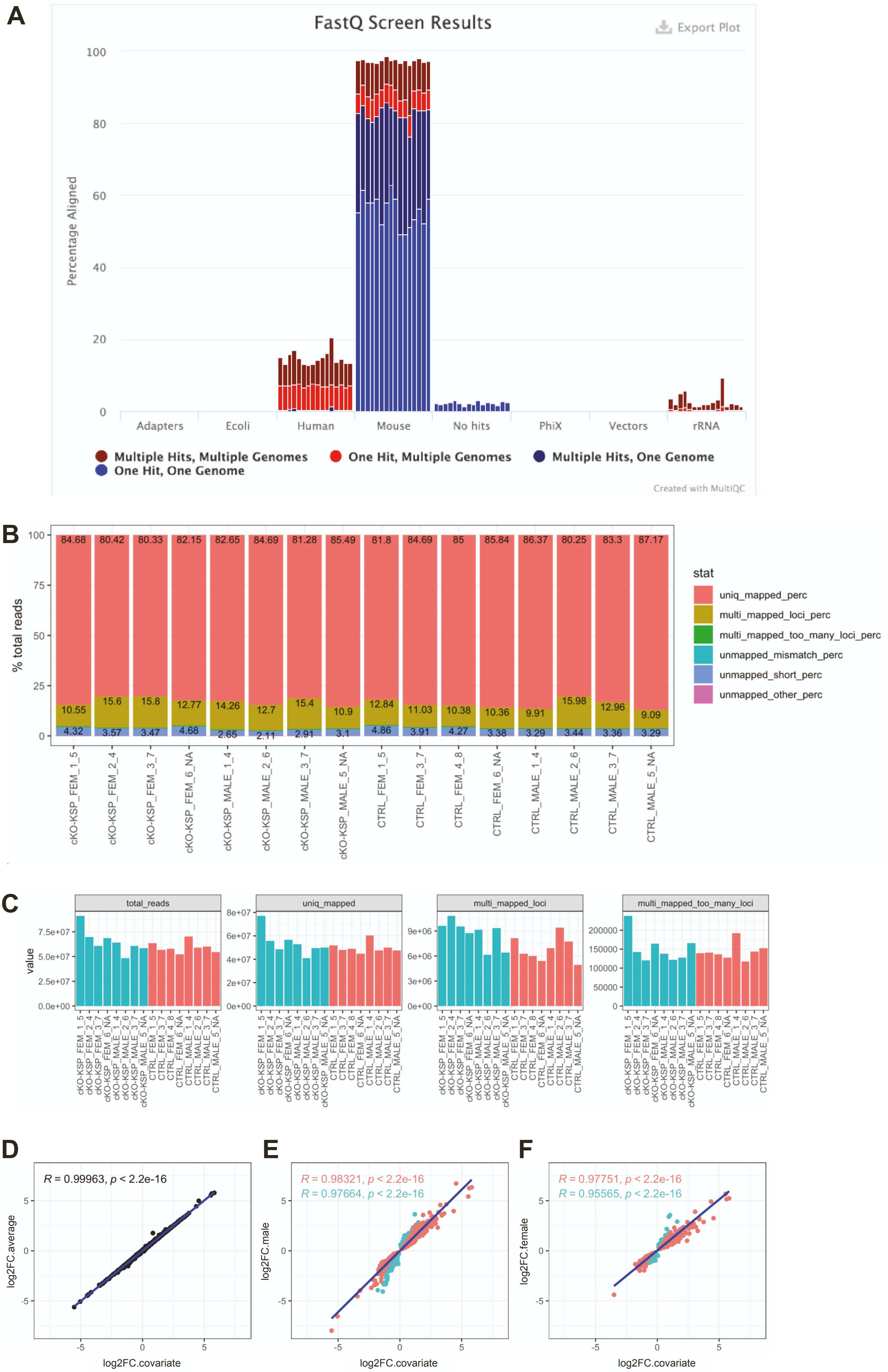
A
FastQ Screen Results
Export Plot
Percentage Aligned
100
80
60
40
20
0
Adapters
Ecoli
Human
Mouse
No hits
PhiX
Vectors
rRNA
Multiple Hits, Multiple Genomes
One Hit, Multiple Genomes
Multiple Hits, One Genome
One Hit, One Genome
Created with MultiQC
B
% total reads
100
75
50
25
0
84.68 80.42 80.33 82.15 82.65 84.69 81.28 85.49 81.8 84.69 85 85.84 86.37 80.25 83.3 87.17
10.55 15.6 15.8 12.77 14.26 12.7 15.4 10.9 12.84 11.03 10.38 10.36 9.91 15.98 12.96 9.09
4.32 3.57 3.47 4.68 2.65 2.11 2.91 3.1 4.86 3.91 4.27 3.38 3.29 3.44 3.36 3.29
cKO-KSP_FEM_1_5
cKO-KSP_FEM_2_4
cKO-KSP_FEM_3_7
cKO-KSP_FEM_6_NA
cKO-KSP_MALE_1_4
cKO-KSP_MALE_2_6
cKO-KSP_MALE_3_7
cKO-KSP_MALE_5_NA
CTRL_FEM_1_5
CTRL_FEM_3_7
CTRL_FEM_4_8
CTRL_FEM_6_NA
CTRL_MALE_1_4
CTRL_MALE_2_6
CTRL_MALE_3_7
CTRL_MALE_5_NA
stat
uniq_mapped_perc
multi_mapped_loci_perc
multi_mapped_too_many_loci_perc
unmapped_mismatch_perc
unmapped_short_perc
unmapped_other_perc
C
value
total_reads
uniq_mapped
multi_mapped_loci
multi_mapped_too_many_loci
7.5e+07
5.0e+07
2.5e+07
0.0e+00
8e+07
6e+07
4e+07
2e+07
0e+00
9e+06
6e+06
3e+06
0e+00
200000
150000
100000
50000
0
D
R = 0.99963, p < 2.2e-16
log2FC.average
log2FC.covariate
E
R = 0.98321, p < 2.2e-16
R = 0.97664, p < 2.2e-16
log2FC.male
log2FC.covariate
F
R = 0.97751, p < 2.2e-16
R = 0.95565, p < 2.2e-16
log2FC.female
log2FC.covariate
5
0
-5

**Figure S8. RNA-seq quality control and alignment metrics for *Bicc1* cKO-Ksp kidneys.** (A) FastQ Screen analysis showing read alignment across reference genomes and sequence categories. The majority of reads aligned to the mouse genome, with minimal contributions from contaminating sequences. Limited off-target alignments were observed, primarily in the human genome. (B) Read mapping summary per sample expressed as percentage of total reads. Across all samples, 80-90% of reads mapped to unique loci, with smaller fractions mapping to multiple loci or remaining unmapped. (C) Absolute read counts per sample for total reads, uniquely mapped reads, and multi-mapped reads. All samples yielded 4-6 × $10^7$ reads per kidney with no overt outliers across genotypes or sexes, indicating consistent sequencing depth and alignment quality. (D) Comparison of genotype-associated log2 fold-change values obtained from the DESeq2 model including sex as a covariate (~ sex + genotype; x-axis) with the averaged sex-stratified log2 fold-change values (y-axis). Each point represents one gene from the complete gene set analyzed. (E-F) Comparison of genotype-associated log2 fold-change values obtained from the covariate model with those obtained from separate male and female analyses, shown in panels E and F, respectively, using genes reported in Table S3. Genes are colored according to significance status, with genes significant only in the corresponding sex-specific analysis shown in blue and genes significant in both the sex-specific and covariate-adjusted analyses shown in pink. Pearson correlation coefficients are shown for each comparison.
